# The Synthetic *Plasmodium falciparum* Circumsporozoite Peptide PfCS102 as a Malaria Vaccine Candidate: A Randomized Controlled Phase I Trial

**DOI:** 10.1371/journal.pone.0007304

**Published:** 2009-10-02

**Authors:** Régine Audran, Floriana Lurati-Ruiz, Blaise Genton, Hildur E. Blythman, Opokua Ofori-Anyinam, Christophe Reymond, Giampietro Corradin, François Spertini

**Affiliations:** 1 Division of Immunology and Allergy, Centre Hospitalier Universitaire Vaudois, Lausanne, Switzerland; 2 Department of Ambulatory Care and Community Medicine, Policlinique Médicale Universitaire, Lausanne, Switzerland; 3 Formerly RMF Dictagene S.A, Lausanne, Switzerland; 4 GlaxoSmithKline Biologicals, Rixensart, Belgium; 5 Institute of Biochemistry, University of Lausanne, Epalinges, Switzerland; Sabin Vaccine Institute, United States of America

## Abstract

**Background:**

Fully efficient vaccines against malaria pre-erythrocytic stage are still lacking. The objective of this dose/adjuvant-finding study was to evaluate the safety, reactogenicity and immunogenicity of a vaccine candidate based on a peptide spanning the C-terminal region of *Plasmodium falciparum* circumsporozoite protein (PfCS102) in malaria naive adults.

**Methodology and Principal Findings:**

Thirty-six healthy malaria-naive adults were randomly distributed into three dose blocks (10, 30 and 100 µg) and vaccinated with PfCS102 in combination with either Montanide ISA 720 or GSK proprietary Adjuvant System AS02A at days 0, 60, and 180. Primary end-point (safety and reactogenicity) was based on the frequency of adverse events (AE) and of abnormal biological safety tests; secondary-end point (immunogenicity) on *P. falciparum* specific cell-mediated immunity and antibody response before and after immunization. The two adjuvant formulations were well tolerated and their safety profile was good. Most AEs were local and, when systemic, involved mainly fatigue and headache. Half the volunteers in AS02A groups experienced severe AEs (mainly erythema). After the third injection, 34 of 35 volunteers developed anti-PfCS102 and anti-sporozoite antibodies, and 28 of 35 demonstrated T-cell proliferative responses and IFN-γ production. Five of 22 HLA-A2 and HLA-A3 volunteers displayed PfCS102 specific IFN-γ secreting CD8^+^ T cell responses. Responses were only marginally boosted after the 3^rd^ vaccination and remained stable for 6 months. For both adjuvants, the dose of 10 µg was less immunogenic in comparison to 30 and 100 µg that induced similar responses. AS02A formulations with 30 µg or 100 µg PfCS102 induced about 10-folds higher antibody and IFN-γ responses than Montanide formulations.

**Conclusions/Significance:**

PfCS102 peptide was safe and highly immunogenic, allowing the design of more advanced trials to test its potential for protection. Two or three immunizations with a dose of 30 µg formulated with AS02A appeared the most appropriate choice for such studies.

**Trial Registration:**

Swissmedic.ch 2002 DR 1227

## Introduction

*P. falciparum* induced malaria is a major cause of disease and death in tropical areas. The difficulty to obtain a protective malaria vaccine is largely related to the genetic complexity of the parasite, to its metamorphosis across several life stages within the human host and to its strategies, acquired with evolution, to escape host immune response. The hope for an efficient vaccine is based on the observation that natural exposure to repeated infections leads to some degree of immune protection and that humans can acquire a complete pre-erythrocytic protection after repeated immunization with irradiated sporozoites (reviewed in [1]). Studies performed in animals and in human volunteers have demonstrated that the anti-sporozoite protection depends on both humoral and cell-mediated immune (CMI) response [2]. Facing the difficulties of the large-scale production of an attenuated sporozoite based vaccine [3], subunit candidates were selected on the basis of the protective immune response obtained with irradiated sporozoites. The circumsporozoite protein (CSP), which is abundant at the cell surface of *Plasmodium* sporozoite, appeared to play a crucial role in preclinical and clinical models of protection [4] implicating humoral [5], CD4^+^ and CD8^+^ T cell response [6]–[14].

Different CSP-derived vaccine constructs have been evaluated in clinical trials, including synthetic peptides, which have the advantage to be rapidly obtainable at high degree of purity and to have absent intrinsic toxicity in clinical grade batches. In preclinical studies, a long synthetic peptide (LSP), encoding the C-terminal 282–383 region of CSP, was found to be highly immunogenic and protective [15]–[18]. In addition, individuals living in malaria-endemic areas develop high titers of specific antibodies and a vigorous CMI response to this sequence [19]. A preliminary Phase I study was completed using a GLP grade P*f* CSP 282–383 batch (PfCS102) [20]. The vaccine was well tolerated and safe. The humoral immune response induced by PfCS102 adjuvanted with Montanide was excellent, as well as the CMI response based on the induction of specific CD8^+^ T cells and IFN-γ secretion.

In view of these results, in this Phase I study we evaluated the safety and immunogenicity of PfCS102 LSP synthesized under GMP conditions, as required for clinical trials, as well as its combination with two adjuvants, AS02A and Montanide, in six groups of healthy adult volunteers for three dose levels of LSP.

## Methods

### Objectives

The objectives of this trial were to investigate the safety and tolerability of the intra-muscular administration of the PfCS102 LSP, injected in combination with two different adjuvants, as a candidate malaria vaccine. We determined the occurrence and severity of vaccine-related adverse events (AE) for each combination of vaccine (primary objective) in order to define the optimal antigen-adjuvant combination, i.e. the combination inducing the highest humoral and CMI response (secondary objective).

### Participants

Protocol and supporting CONSORT Checklist are available as supporting information; see Checklist S1 and Protocol S1. Volunteers, male and female between 18 and 45 years, were recruited in the Lausanne area, Switzerland, and examined at the Centre Hospitalier Universitaire Vaudois, Vaccination and Immunotherapy Center. Volunteers should not have lived in endemic areas, were negative for HIV, HBV and HCV antibody screening, and were excluded when presenting with any acute or chronic medical conditions, evidence of abnormality in clinical or laboratory findings, pregnancy, previous history of malaria, or positive antibody response to PfCS102 by ELISA. During the study period, volunteers were not authorized to visit or to reside in a malaria endemic area. Female participants had to take an adequate contraception. A urinary pregnancy test was done before injections. After inclusion, participants were tested for HLA locus A by PCR-SSO (LiPA-HAL-A Update, Innogenetics, Ghent, B) according to manufacturer's instruction, in preparation of the monitoring of PfCS102 specific MHC class I responses in HLA-A2 and -A3 volunteers for whom PfCS epitopes were known.

### Ethics Statement

The protocol was approved by the Ethical Review Board of the Faculty of Biology and Medicine, Lausanne, Switzerland and by the Swiss Regulatory Agency For Therapeutic Products (Swissmedic, #2002 DR 1227); the approvals are available as supporting information, Approval letters S1 and S2. Before enrolment into the study, all volunteers gave their written informed consent. A copy of the consent form is available as supporting document, Consent form S1. The trial was conducted according to GCP and the principles of The Declaration of Helsinki.

### Vaccine preparation

The vaccine LSP PfCS102 (KNNQGNGQG HNMPNDPNRN VDENANANSA VKNNNNEEPS DKHIKEYLNK IQNSLSTEWS PCSVTCGNGI QVRIKPGSAN KPKDELDYAN DIEKKICKME KCS) encompassed the C-terminal non-repeat region 282-383 of CSP of the *P.falciparum* strain NF54. The peptide was synthesized, purified, bottled and lyophilized according to GMP procedures (batch 01FS019, RMF Dictagene SA, Epalinges, Switzerland) and tested for stability and toxicity (sterility, endotoxin content, apyrogenicity) as well as for immunogenicity in mice when formulated with adjuvants cited below.

Peptide-adjuvant combinations were prepared under sterile conditions within four hours of injection. PfCS102 as a lyophilized powder was dissolved at room temperature either (A) in 180 µl of sterile NaCl 0.9% (Bichsel AG, Interlaken, Switzerland), mixed with 420 µl of Montanide ISA-720 (Seppic Paris, France) and passed at least 10 times through a 21G needle to form a stable (3/7, w/w) water-in-oil emulsion according to manufacturer's instructions, or (B) directly in 600 µl of GSK proprietary Adjuvant System AS02A (oil-in-water emulsion with the immunostimulants monophosphoryl lipid A (MPL; GSKBio NA, WA,USA) and *Quillaja saponaria* fraction 21 (QS21; Antigenics, New York, NY, USA). For 10 and 30 µg doses, peptide was first diluted in NaCl 0.9% or in AS02A.

Whatever the peptide dose (10, 30 and 100 µg), the final quantity of adjuvant Montanide ISA-700 or AS02A for each formulation was constant.

### Phase I study design

This study was designed as a randomized, double-blind, dose-ranging study to assess the safety and immunogenicity of three vaccine dose regimens combined with two different adjuvants. Six groups of 6 subjects received 3 injections at days 0, 60 and 180, of 10, 30 or 100 µg of peptide in Montanide ISA 720 (groups M10, M30, M100 respectively) or in AS02A (groups A10, A30 and A100). The vaccine was delivered as a 500 µl injection intramuscularly in the deltoid region of alternating arm.

At days 0, 30, 60, 75, 180, 195 and 360, whole blood samples were collected on citrate as anticoagulant (Vacutainer CPT, BD, Basel, Switzerland); peripheral blood mononuclear cells (PBMC) were separated on a density gradient, used fresh in proliferation assay or stored in liquid nitrogen for other analysis. Plasma was collected after the first centrifugation.

### Assessment of safety and tolerability

Volunteers were observed for one hour after each immunization and returned for a follow-up visit 2 and 30 d after the first injection, 2 and 15 d after the second injection and 2, 15, 180, and 360 d after the third injection. Safety parameters included clinical observations and laboratory measurements. Solicited local and general AEs were monitored at each follow-up visit and self-reported in personal diaries from day 0 to 3 after each injection. Unsolicited local and general AEs were monitored up to 30 d after injection in diary cards, but had to be reported anytime during the whole trial at each visit. Diary cards were collected at days 30, 75 and 195 and furthermore a phone call was made by the investigator 30 d after the 2nd and 3rd injection to obtain an update on new or previously ongoing AEs. Subjects were requested to come to the site for unresolved AEs related to the vaccination Serious AEs were recorded throughout the study. Solicited local AE included pain, skin erythema, pruritus, induration (including swelling) and functional limitation of the arm. Solicited systemic AEs included fatigue, headache, fever, arthralgia, myalgia, gastro-intestinal disorders, exanthema, urticaria, dyspnea, fainting, dizziness, sweating and palpitations. AEs (solicited and unsolicited) were graded from 0 to 3 (grade 0: no impairment; grade 1 (mild): easily tolerated; grade 2 (moderate): interference with daily activity; grade 3 (severe): prevention of daily activities), except for erythema and induration, which were graded on the size of the lesion measured by the investigator 48 h after injection (grade 1 (mild): 1–19 mm; grade 2 (moderate): 20–49 mm; grade 3 (severe): ≥50 mm) and fever, which was graded on a scale of axillary temperature (grade 0: <37,5°C; grade 1: 37.5–37.9°C; grade 2: 38–38.9°C;. grade 3:≥39°C). Subjects were withdrawn from receiving further vaccinations if they were presenting a local induration after the 1^st^ or the 2^nd^ injection measuring ≥12 cm in diameter. Haematology (hemoglobin, hematocrit, white blood cell count, platelet count) and blood chemistry parameters (blood urea nitrogen, serum creatinine, alanine aminotransferase, aspartate aminotransferase, alkaline phosphatase) were evaluated at screening, 30 d after the first injection, 15 d after the second injection and 15 and 180 d after the third injection.

### Peptides

In proliferation assay, three overlapping peptides spanning 100% of PfCS102 were used to identify most immunogenic regions: PfCS 282–318 (KNNQGNGQG HNMPNDPNRN VDENANANSA VKNNNNEE), PfCS 319–383 (PS DKHIKEYLNK IQNSLSTEWS PCSVTCGNGI QVRIKPGSAN KPKDELDYAN DIEKKICKME KCS) and PfCS 310–340 (A VKNNNNEEPS DKHIKEYLNK IQNSLSTEWS). MHC-class I (MHC-I) restricted cytotoxic T lymphocyte (CTL) epitopes were used in CD8^+^ T cell stimulation assays and prior to intracellular cell staining (ICS) (HLA-A2 specific epitopes: PfCS 327–335 (YLNKIQNSL), PfCS 292–301 (NMPNDPNRNV); HLA-A3 specific epitopes: PfCS 344–342 (VTCGNGIQVR), PfCS 353–361 (RIKPGSANK)) as well as peptide controls FLU MP 58–66, (GILGFVFTL), CMV pp65 495–503 (NLVPMVATV) and FLU NP 265–273 (ILRGSVAHK).

### Antibody responses

Anti-PfCS102 antibodies were measured in plasma samples by ELISA as previously described [20]. Results were expressed as arbitrary units (U/ml) according to a linear standard curve, 100 U/ml corresponding to a titer of 1/12800. ELISA was performed blind by the lab technician, then validated and interpreted blind by lab technician and scientific supervisor. The presence of antibody recognizing *P. falciparum* CS protein in its native configuration was determined by an indirect IFA method [20] using NF54 parasites fixed on 10-well slides, 5000 sporozoites/well (kindly provided by C. C. Hermsen, NL-Nijmegen). For IFA, data was based on the evaluation of two blinded examiners. Results were expressed as geometric means of titrations defined as the last plasma dilution giving a positive signal. A response above the baseline, defined as the mean response of pre-immune sera + 3SD, was considered positive.

### T cell proliferation assay and cytokine measurements

PBMC were stimulated with PfCS102 (1.2, 6 and 30 µg/ml) or with tetanus toxoid (TT, 10 µg/ml) or phytohemagglutinin (PHA, 5 µg/ml) as controls, as previously described [21]. Production of IFN-γ and IL-10 in supernatants of five-day proliferation cultures was measured by ELISA (Elipair, Diaclone, France), following manufacturer's instructions and expressed as pg/ml. Experimental median lower limits of detection of IFN-γ, IL-10 were 0.74, 0.45 pg/ml respectively. The specific activity of IFN-γ kit standard was titrated against NIBSC IFN-γ European standard and was set at 3.61 IU for one ng IFN-γ. A response above the baseline, defined as the mean response of pre-immune cell sample proliferation + 3SD, was considered positive.

### Antigen-specific CD8 T cell detection by long-term ELISPOT

Cryopreserved PBMC from HLA-A2 volunteers collected before the first immunization and after the second and the third immunization were thawed simultaneously. CD8^+^ T cells were purified by magnetic cell sorting (Miltenyi Biotec, D-Bergish-Glabach) and cultured in the presence of 1 µM CTL peptides (PfCS or controls) and irradiated autologous CD8^−^ cells. On days 9 to 12 of culture, the frequency of antigen specific CD8^+^ T cell activity was evaluated by IFN-γ ELISPOT as described [7]. Spots were counted using a computerized system (BioSys GmbH, D-Karben).

### Intracellular cytokine staining

The frequency of PfCS102 specific CD4^+^ or CD8^+^ T cells was evaluated by ICS using PBMC of HLA-A2 and/or HLA-A3 volunteers. Pre- and post-immunization PBMC were tested simultaneously. PBMC (6×10^6^ cells/ml) in culture medium were stimulated for 18 to 20 hours with peptides (PfCS102, 10 µg/ml, plus HLA-A2 and/or HLA-A3 CTL peptides, 5 µg/ml each) in the presence of brefeldin A (10 µg/ml, Sigma) during the last four hours of culture. Unstimulated or SEB (3 µg/ml) stimulated cells served as controls. To detect activated cells, PBMC were fixed (FACS lysing buffer, BD), permeabilized (Perm 2, BD) and stained with anti-IFN-γ, -FITC (clone B27), anti-CD4-PerCP-Cy5 (clone SK3) from BD and anti-CD8-RPE (clone DK25, Dako, DK-Glostrup) antibodies. Additional analysis of CD8^+^ cell subsets were determined by staining with anti-IFN-γ, -FITC, anti-CD3-PerCP-Cy5 (clone B27), anti-CD56-APC (clone B159) (BD), anti-CD8-RPE (Dako) and anti-CD16-APC (clone 3G8, Serotec). Frequency of IFN-γ secreting lymphocytes in the selected populations was expressed using the formula: (% IFN-γ positive cells in stimulated cells – % IFN-γ positive cells in unstimulated cells). A response above the baseline, defined as the mean frequency of pre-immune cell samples + 3SD, was considered positive.

### Sample size

This phase I study was designed as a double blind study to evaluate safety and immunogenicity in a descriptive *per* total vaccinated subjects' analysis. Sample size was thus not defined on the power of the statistical analysis. However a number of 18 subjects in stratification per adjuvant and 12 subjects in stratification per peptide doses were estimated reasonably large for non-parametric statistical analysis.

### Randomization

Subjects enrolled by the clinical investigator were randomized by the statistician per antigen dose, by blocks of 12 subjects, 6 (3 males, 3 females) to each adjuvant cohort. Immediately after screening and recruitment, the statistician provided randomization lists to the pharmacist in charge of vaccine preparation and randomization envelopes to the principal investigator. Vaccinations started with the 10 µg block. After review of safety data collected for at least 21 days between dose block, the 30 µg block was vaccinated and finally the 100 µg block. One withdrawal that occurred before the 2^nd^ injection was replaced with a subject of the same gender from the list of screened volunteers.

### Blinding

Study participants and investigators who assessed outcomes were blinded to vaccine adjuvant assignment. The trial could not be blind for peptide dose because of the dose-escalating design. Vaccines were prepared by a pharmacist team who had no contact with participants. Because of the difference in visual aspect and in viscosity between adjuvants, a nurse performed the injections in a separate room, in the absence of the clinical investigator. Study codes were broken after study completion.

### Statistical evaluation

Statistical analysis of clinical response (safety and reactogenicity) was descriptive and *per* total vaccinated subjects. When indicated, two-sided statistical tests were performed regarded as exploratory. Differences between adjuvant or dose groups were analyzed by Fisher's exact test for safety and reactogenicity data (numbers of AEs). For immunological data, comparisons of adjuvant groups were performed using a Kruskal-Wallis Rank-Sum test on the log of the ratio of the “per time” values on those at D0 (SAS software). Other comparisons of immunological data including CD8^+^ and CD4^+^ T cell responses were calculated using non parametric Wilcoxon or Mann-Whitney t tests for paired within-group and between-groups comparisons respectively (GraphPad Prism software). Data were considered significant when *p* was <0.05.

## Results

### Participant flow chart (fig. 1)

At the screening visit, 52 volunteers were examined and subsequently 15 were immediately excluded: in 8 cases for abnormalities in the clinical safety tests, in 3 cases for positive anti-PfCS102 antibodies, in 1 case for consent withdrawal, in 2 cases for recent travel in a malaria endemic country, and in 1 case for abnormal cardiovascular physical exam. Globally, 37 healthy adults were randomized. One volunteer from group M10 was withdrawn after the first injection for a non vaccine related severe adverse event (AE) and was replaced. Thirty-six volunteers received 2 injections. Thirty-five of them received the three scheduled injections. The study report includes clinical and immunological data from 37 volunteers (19 in the Montanide ISA 720 adjuvant group and 18 in the AS02A adjuvant group) up to visit 12 (day 540).

**Figure 1:**
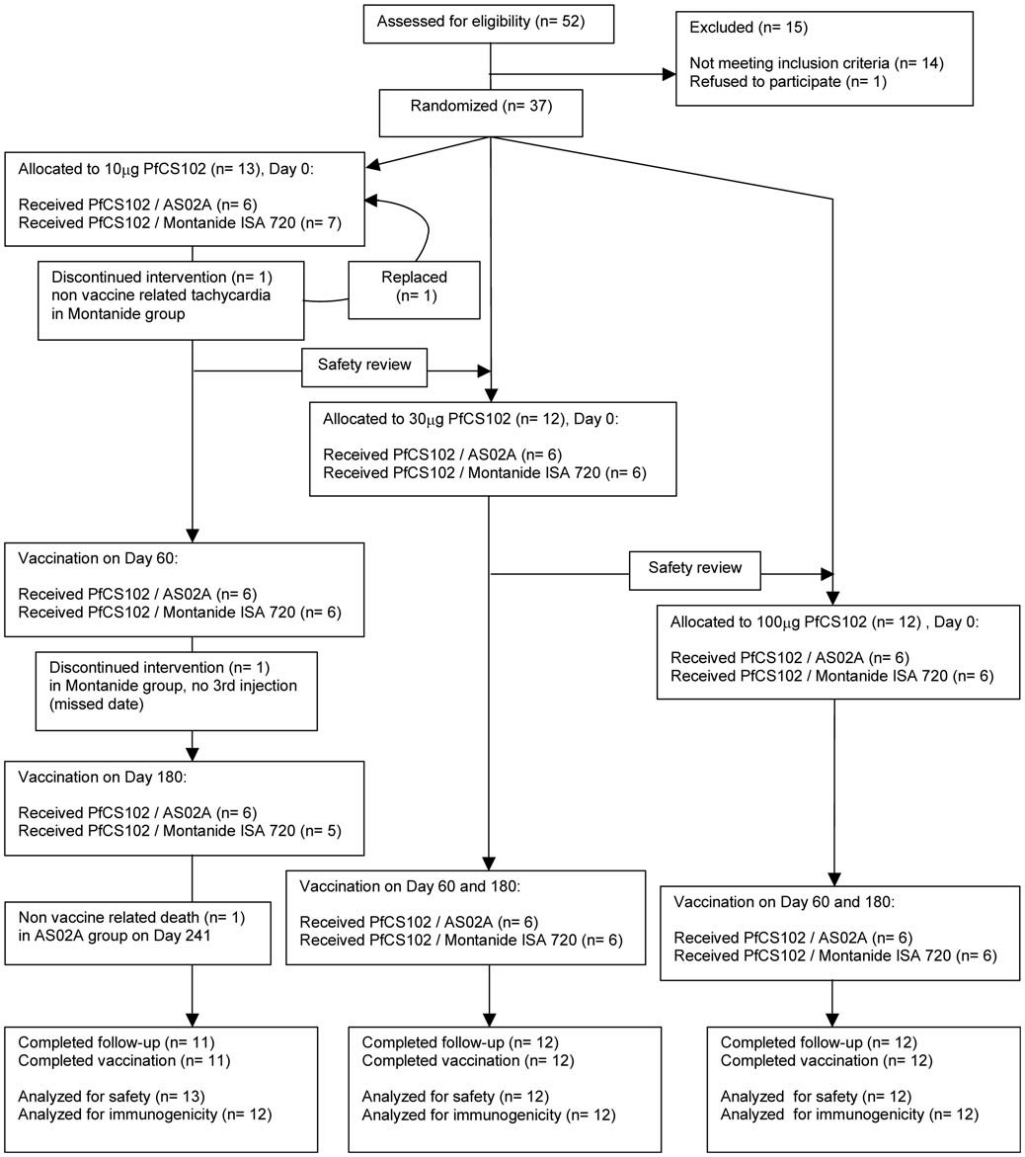
Study flow chart. Design of the Phase I, double-blind, dose-ranging study of 3 vaccine dosing regimens combined to two different adjuvants AS02A and Montanide ISA 720. For each dose, volunteers were randomized by blocks of 12, with 6 individuals per adjuvant. Volunteers were seen 2 and 30 d after the first injection, 2 and 15 d after the second injection and 2, 15, 180, and 360 d after the third injection. A phone call was addressed to all volunteers at d 30 after the 2^nd^ and 3^rd^ injection.

### Recruitment

Recruitment and enrollment occurred from December 2^nd^, 2002 to May 20^th^, 2003. Study duration was 18 months and the last visit occurred on November 24^th^, 2004.

### Baseline data

Baseline safety laboratory values were all within the normal range (not shown). There was no imbalance between age, sex and baseline clinical safety tests at enrolment between the various groups (Table 1).

**Table 1:** Characteristics of participants in various immunization groups.

Groups	n total	n female	n HLA-A2 and/or -A3	Age in years mean (95% CI)
**10 µg PfCS102 in AS02A**	6	3	3	30.2 (8.4)
**30 µg PfCS102 in AS02A**	6	3	3	29.0 (4.1)
**100 µg PfCS102 in AS02A**	6	3	3	25.7 (3.2)
**10 µg PfCS102 in M ISA 720**	7	4	4	25.0 (5.5)
**30 µg PfCS102 in M ISA 720**	6	3	4	28.4 (5.8)
**100 µg PfCS102 in M ISA 720**	6	3	5	30.8 (7.5)

### Assessment of safety and tolerability

Occurrences of local and systemic reactions imputable to vaccine after each injection are described in Table 2 and 3.

**Table 2:** Local adverse events during the 4-day follow-up after each immunization.

	Group			Any symptom	Pain	Erythema	Pruritus	Induration	Functional limitation	Any Gr.3
	Adjuvant	PfCS dose (µg)	n		Overall (Gr.3)	Overall (Gr.3)	Overall (Gr.3)	Overall (Gr.3)	Overall (Gr. 3)	
***Injection 1***	**A**	**10**	6	**6**	**6** (**1**)	**3** (**3**)	**1** (0)	**4** (**1**)	**4** (**1**)	**3**
		**30**	6	**6**	**6** (0)	**2** (**2**)	**1** (0)	**3** (**1**)	**5** (0)	**1**
		**100**	6	**5**	**5** (0)	**2** (**2**)	**1** (0)	**1** (0)	**5** (0)	**2**
	**M**	**10**	7	**5**	**4** (0)	**1** (**1**)	**2** (0)	**3** (0)	0 (0)	**1**
		**30**	6	**4**	**4** (0)	0 (0)	0 (0)	0 (0)	**2** (0)	0
		**100**	6	**4**	**4** (0)	0 (0)	0 (0)	**1** (0)	**2** (0)	0
***Injection 2***	**A**	**10**	6	**6**	**6** (**1**)	**4** (**4**)	**1** (0)	**2** (**1**)	**4** (0)	**4**
		**30**	6	**6**	**6** (0)	**5** (**3**)	**1** (0)	**5** (**2**)	**3** (**1**)	**4**
		**100**	6	**6**	**6** (**1**)	**2** (**2**)	**1** (0)	**3** (**1**)	**5** (0)	**3**
	**M**	**10**	6	**5**	**4** (**1**)	**2** (0)	0 (0)	**4** (0)	**2** (0)	**1**
		**30**	6	**5**	**5** (0)	**2** (0)	**1** (0)	**1** (0)	**3** (0)	0
		**100**	6	**5**	**5** (**1**)	**2** (**2**)	0 (0)	**1** (0)	**3** (**1**)	**2**
***Injection 3***	**A**	**10**	6	**6**	**5** (0)	**3** (**3**)	0 (0)	**1** (**1**)	**3** (0)	**3**
		**30**	6	**6**	**6** (0)	**3** (**3**)	**1** (0)	**2** (**2**)	**4** (0)	**3**
		**100**	6	**6**	**6** (0)	**5** (**4**)	**1** (0)	**3** (**1**)	**6** (0)	**4**
	**M**	**10**	5	**5**	**4** (0)	**1** (0)	0 (0)	**2** (0)	**1** (0)	0
		**30**	6	**4**	**4** (0)	**1** (0)	**1** (0)	**1** (0)	**1** (0)	0
		**100**	6	**5**	**4** (0)	0 (0)	0 (0)	**2** (0)	**4** (0)	0

Data are numbers of participants presenting with symptoms. n: number of participants per analysis group. M: Montanide ISA 720. A: AS02A. Gr.3: grade 3.

**Table 3:** Solicited systemic adverse events during the 4-day follow-up after each immunization.

	Group			Any symptom	Fatigue	Headache	Fever	Arthralgia	Myalgia	GI	Other§	Any Gr. 3
	Adjuvant	PfCS dose (µg)	n		Overall (Gr.3)	Overall (Gr.3)		Overall (Gr.3)	Overall (Gr.3)	Overall (Gr.3)		
***Injection 1***	**A**	**10**	6	**3**	**3** (**1**)	**2** (0)	0	0 (0)	**1** (0)	**1** (0)	0	**1**
		**30**	6	**4**	**3** (0)	**1** (0)	0	0 (0)	**1** (0)	**1** (0)	0	0
		**100**	6	**4**	**4** (0)	**3** (0)	**1**	**4** (0)	**4** (0)	0 (0)	**1**	0
	**M**	**10**	7	**3**	**3** (0)	0 (0)	0	**1** (0)	0 (0)	**1** (**1**)	**2**	**1**
		**30**	6	**4**	**3** (**1**)	**4** (0)	0	0 (0)	0 (0)	**1** (0)	**1**	**1**
		**100**	6	**3**	**1** (0)	**2** (0)	0	0 (0)	0 (0)	0 (0)	**2**	0
***Injection 2***	**A**	**10**	6	**4**	**3** (**1**)	**2** (**1**)	0	**1** (0)	**2** (0)	**1** (0)	**1**	**2**
		**30**	6	**4**	**3** (0)	**1** (0)	0	0 (0)	0 (0)	0 (0)	**2**	0
		**100**	6	**4**	**3** (0)	**3** (0)	0	**1** (0)	**1** (0)	0 (0)	**1**	0
	**M**	**10**	6	**1**	**1** (0)	0 (0)	0	**1** (**1**)	0 (0)	0 (0)	0	**1**
		**30**	6	**4**	**3** (**1**)	**2** (0)	0	0 (0)	0 (0)	**1** (0)	0	**1**
		**100**	6	**3**	**1** (0)	**2** (0)	0	**1** (0)	0 (0)	0 (0)	**1**	0
***Injection 3***	**A**	**10**	6	**3**	**3** (**1**)	**2** (0)	0	0 (0)	0 (0)	**2** (0)	**1**	**1**
		**30**	6	**3**	**3** (**1**)	**1** (0)	**1**	**1** (**1**)	**2** (**1**)	**1** (0)	0	**1**
		**100**	6	**5**	**5** (**1**)	**4** (**1**)	**2**	**4** (**1**)	**3** (**1**)	0 (0)	**3**	**1**
	**M**	**10**	5	**1**	**1** (0)	0 (0)	0	0 (0)	0 (0)	0 (0)	**0**	0
		**30**	6	**2**	**1** (0)	**1** (0)	0	0 (0)	0 (0)	0 (0)	0	0
		**100**	6	**2**	**1** (0)	**1** (0)	0	0 (0)	**1** (0)	**1** (0)	0	0

Data are numbers of participants presenting with symptoms. n: number of participants per group. M: Montanide ISA 720. A: AS02A. GI: gastrointestinal troubles. Gr.3: grade 3.

§ Other solicited adverse events included dizziness (n = 6), sweating (n = 5), dyspnea (n = 3), exanthema, palpitations, hand erythema (n = 1).

### Solicited AE

AEs were mainly local. No local AE considered as unacceptable (induration >12 cm in diameter) occurred in any group. After the first injection, a higher number of AEs occurred in the AS02A (A) adjuvant group than in the Montanide ISA 720 (M) group (17/18 vs 13/19 respectively). This tendency was significant for pain (*p* = 0.042), erythema (*p* = 0.0188), and functional limitation (*p* = 0.0009). The same trend was observed when intensity of all adverse reactions was considered (6/18 grade 3 AEs in A group *vs* 1/19 in M group). Similarly, after the second and third injection, there was a significantly higher occurrence of erythema (*p* = 0.0275 and 0.0045 respectively) and grade 3 AEs (11/18 in A group *vs* 3/18 in M group (*p* = 0.0153), and 10/18 in A group *vs* 0/17 in M one respectively (*p* = 0.0003)). Most severe local AEs (n = 47) resolved within 48 h (23/47), others within 72 h (13/47), within 96 h (6/47) and >96 h (5/47) and consisted in majority (28/47) in erythema. Besides functional limitation which increased together with dose increase, the frequency of other AE did not appear to be affected by peptide dose after any of the three injections.

Overall, systemic adverse reactions were dominated by fatigue (n = 45) and headache (n = 31), followed by myalgia (n = 15) and arthralgia (n = 13), and tended to be more frequent in the AS02A group (n = 34) than in the Montanide ISA 720 group (n = 23). After the first injection, 6 volunteers in the A group presented with myalgia, but none in the M group (*p* = 0.008). After the second injection, there was not much difference between adjuvant groups, but after the third injection, frequency of fatigue and arthralgia was higher in the A group than in the M group (11/18 in A, 3/17 in M and, 5/18 in A and 0/17 in M respectively). There was a slight trend towards an increase in the frequency of systemic AE with increasing peptide dose but this was statistically significant only for arthralgia (*p* = 0.0455).

### Unsolicited AE

Only “probably vaccine related” AEs were reported. They started during the 4-day follow-up after injection, were mostly systemic and consisted in adenopathies (n = 2), shivers (n = 4), pharyngitis (n = 1) and insomnia (n = 1). All were associated with, at least, fatigue, headache, myalgia, local pain and functional limitation, and all occurred in AS02A groups.

### Serious and unexpected AE

A single severe AE (5 minute episode of palpitations) occurred 4 hours after the first injection during physical stress. On the basis of the electrocardiogram, the volunteer was suspected of a short PQ syndrome and referred to a cardiologist for follow-up. The AE was considered as unrelated to the vaccine but led to volunteer withdrawal from further injections. Three serious adverse events occurred between day 195 and day 540; none of them were considered vaccine-related: in A10 group, a car accident with fatal issue at day 241, and in M10 group, a voluntary interruption of pregnancy (14 months after the 2^nd^ vaccination, 3^rd^ injection was not performed) and the surgical removal of a sacrococcygeal dermoid cyst at day 384.

### Laboratory parameters

Hematology as well as blood chemistry test results performed during the injection and follow-up periods were within the normal range, apart from one case of mild alkaline phosphatase increase above the normal range (36–108 U/l), at days 75, 195 and 360 (121, 129, 150 U/l, respectively), not related to clinically significant abnormality. The volunteer was later referred to his general practitioner.

### Anti-PfCS102 antibody response

**Figure 2:**
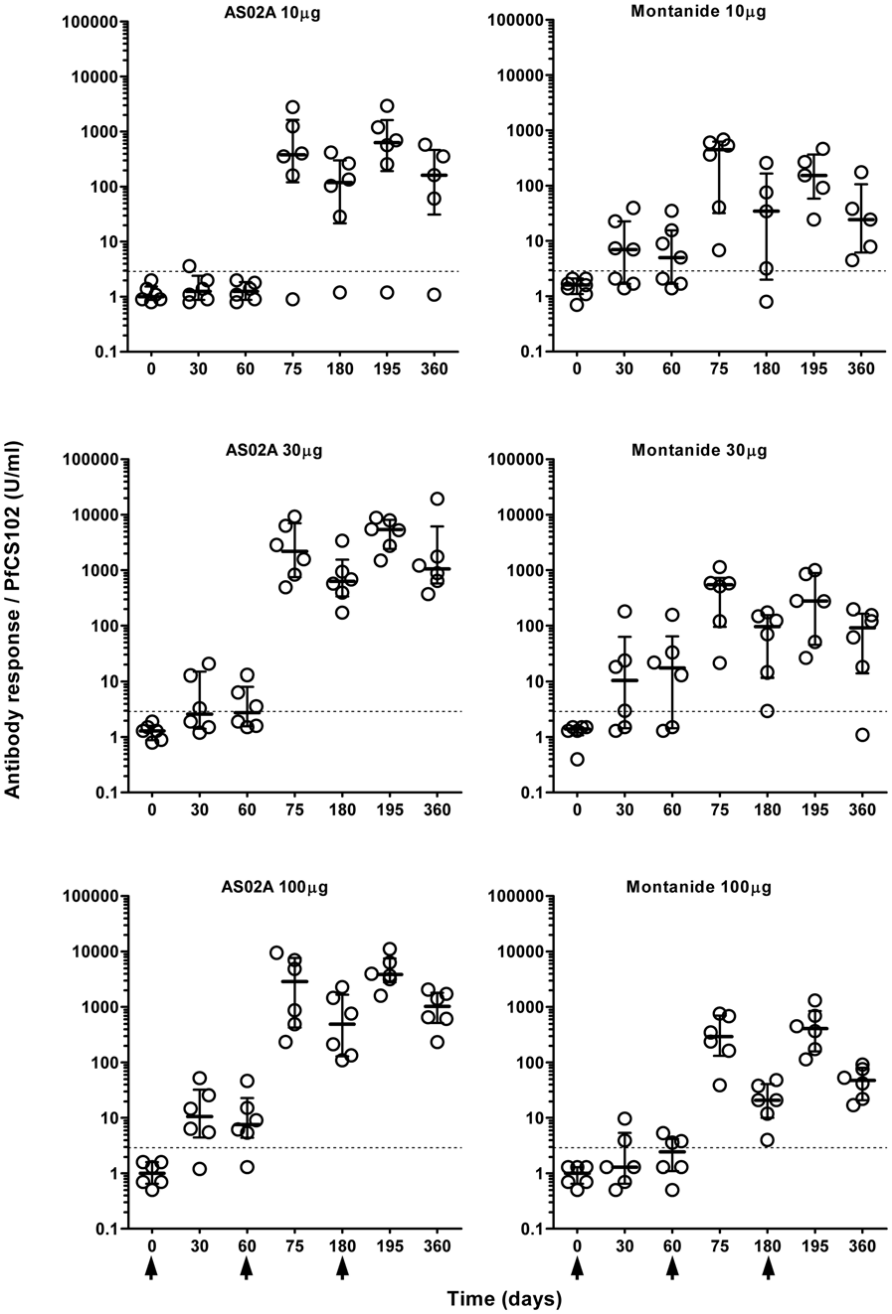
Time course of anti-PfCS102 antibody response upon PfCS102 immunization. Anti-PfCS102 antibodies were measured in plasma samples by ELISA. Results are expressed as individual data in U/ml for each immunization group, with quartiles. Arrows indicate the times of vaccination.

Two weeks after the second and the third injection, 97% of immunized volunteers (35/36 and 34/35 respectively) produced antibodies against PfCS102 above the baseline (2.9 U/ml) (medians and quartiles of all responses: 559 U/ml [237.5; 1162.9] and 695.9 U/ml [262.8; 3047.8] respectively) (Figure 2). At day 360, 94% (32/34) of volunteers still displayed a positive response although of decreasing intensity (median  = 157.9 U/ml [39.0; 636.6]. The level of response was largely higher than the response obtained in the preliminary PfCS102 study (maximum163 U/ml, titer of 25600) [20] or than those observed in endemic area (median 17.8 U/ml [6.1; 55.5], n = 6). Frequencies of responders were similar among all six groups. Nevertheless, after the 2^nd^ and the 3^rd^ immunization, volunteers who received AS02A developed the highest levels of anti-PfCS102 antibodies without differences between the 30 µg and 100 µg doses, but a significantly lower response with the 10 µg dose. In contrast, no dose effect was observed with Montanide ISA 720. Overall, pooling results of doses 30 µg and 100 µg, antibody levels were at each time-points higher in the A group than in the M group, and significant at days 180, 195, 360 (*p*<0.004, Kruskal-Wallis Rank-Sum test). At day 360, levels of antibody responses were at least one log higher in groups immunized with 30 µg or 100 µg PfCS102 formulated with AS02A than with Montanide ISA 720 (*p* = 0.0065 and 0.0039, respectively, Kruskal-Wallis Rank-Sum test).

### Anti-sporozoite responses

**Figure 3:**
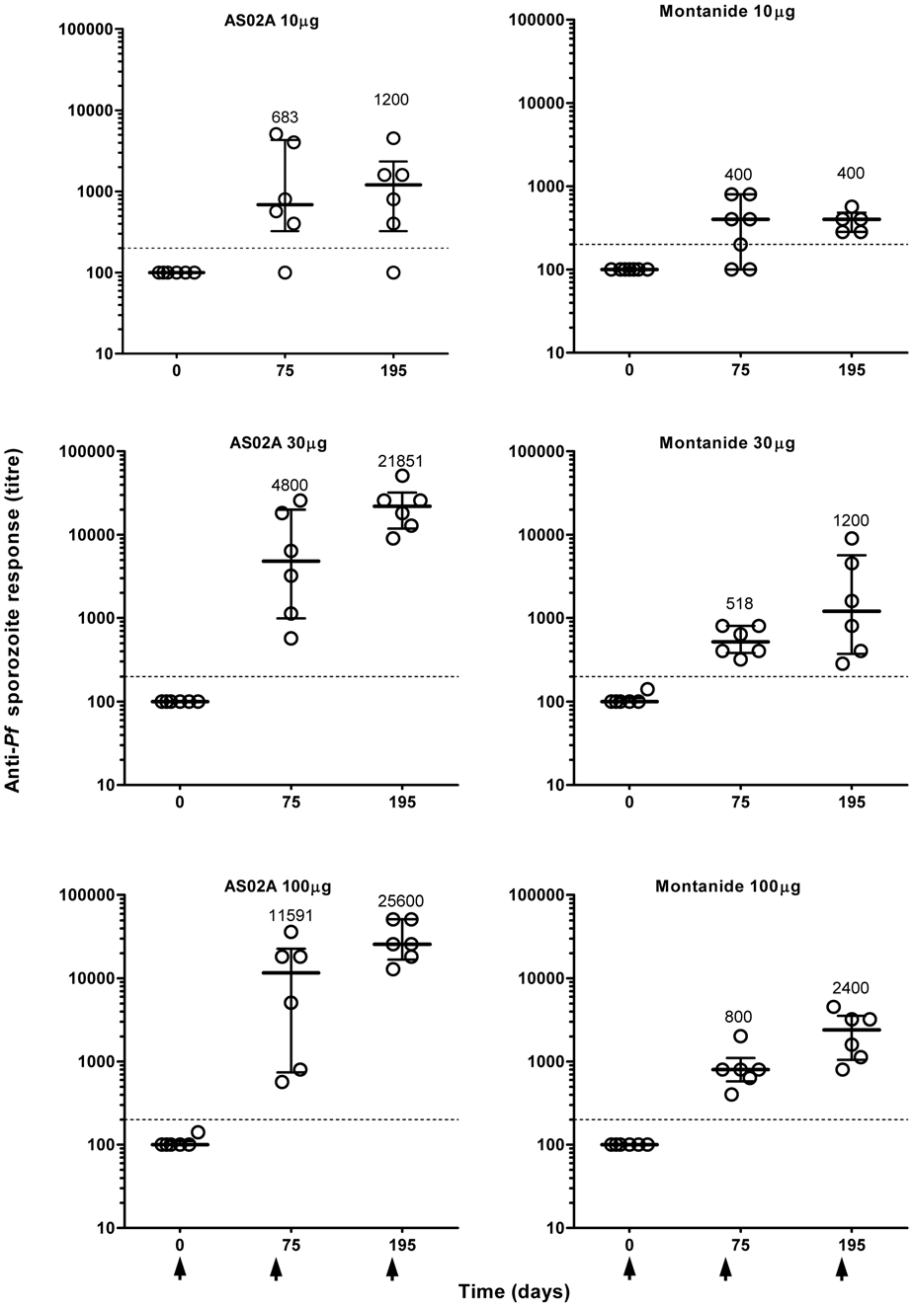
Time-course of anti-*Plasmodium falciparum* antibody response upon PfCS102 immunization. Anti-parasite antibodies were measured in plasma samples by immunofluorescence. Results are expressed as individual titers for each immunization group, with quartiles. Arrows indicate the times of vaccination.

At day 0, none of the volunteers (n = 37) displayed antibodies specific for *P. falciparum* sporozoites (median titer  = 100 (range 100 to 141) (Figure 3). Anti-sporozoite responses with titers ≥200 were considered positive. After the second injection, at day 75, 94% (34/36) of volunteers developed a positive anti-parasite response with a median titer of 800 (quartiles 400–3408) (geometric mean titer: 1199), and after the third injection, 97% (34/35) (median titre: 3200, quartiles 683–15451; geometric mean titer: 2956).

Whatever the adjuvant, the lowest responses were associated with the 10 µg PfCS102 dose. Injections with the 30 µg and 100 µg doses ended up with the same level of responses, as observed above for anti-PfCS102 responses measures by ELISA. The 3^rd^ injection did not improve significantly the responses obtained after the second immunization, except for group M100 (*p* = 0.0312, Wilcoxon's test). AS02A formulations elicited significantly higher responses (10-fold increase) than Montanide ISA 720 using a dose of 30 µg (*p* = 0.0011 at D75, *p*<0.0001 at D195) or 100 µg of peptide (*p* = 0.0189 at D75, *p*<0.0001 at D195, Kruskal-Wallis Rank-Sum test).

### T cell proliferation

**Figure 4:**
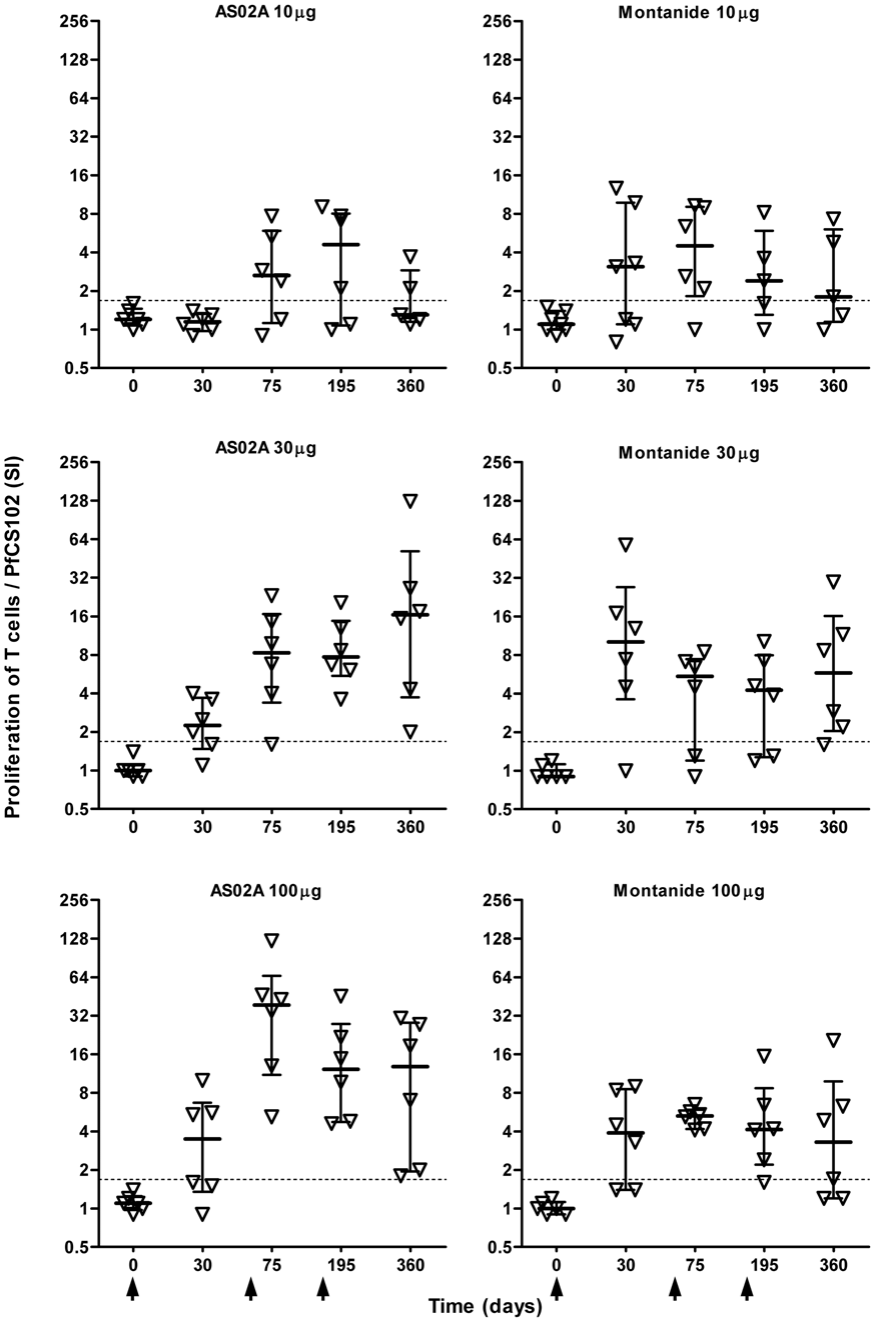
Time-course of anti-PfCS102 proliferative T cell response. Proliferative responses were evaluated by ^3^[H]-thymidine incorporation by peripheral blood mononuclear cells (PBMC) upon stimulation with antigen. Results are expressed as individual stimulation indices (SI) in each immunization group with quartiles. Arrows indicate the times of vaccination.

The first immunization with at least 30 µg PfCS102 induced significant responses that were boosted by the 2^nd^ immunization, in AS02A groups, but not by the third immunization (Figure 4). After the 2^nd^ and 3^rd^ immunization, the intensity of the response (median SI: 5.4 (quartiles 2.5 and 9.0)) increased above the baseline (SI  = 1.64) in 32 out of 36 volunteers (89% responders), 15/18 and 15/18 at D75 and, 16/18 and 12/17 at D195 in AS02A and Montanide ISA 720 groups respectively. The dose of 30 µg was sufficient to induce maximal responses. In term of frequency of responders, there was no difference between the two adjuvants. However, responses obtained after the second immunization with the dose of 100 µg formulated with AS02A were stronger than with Montanide ISA 720 (*p* = 0.026, Mann-Whitney's test). Six months after the last immunization (day 360), 26 out of 34 volunteers were still responders. For all volunteers, proliferative responses to the shorter CS 310–340 and 310–383 peptides were comparable to those obtained with PfCS102, the CS 282–318 inducing only weak responses.

### IFN-γ response

**Figure 5:**
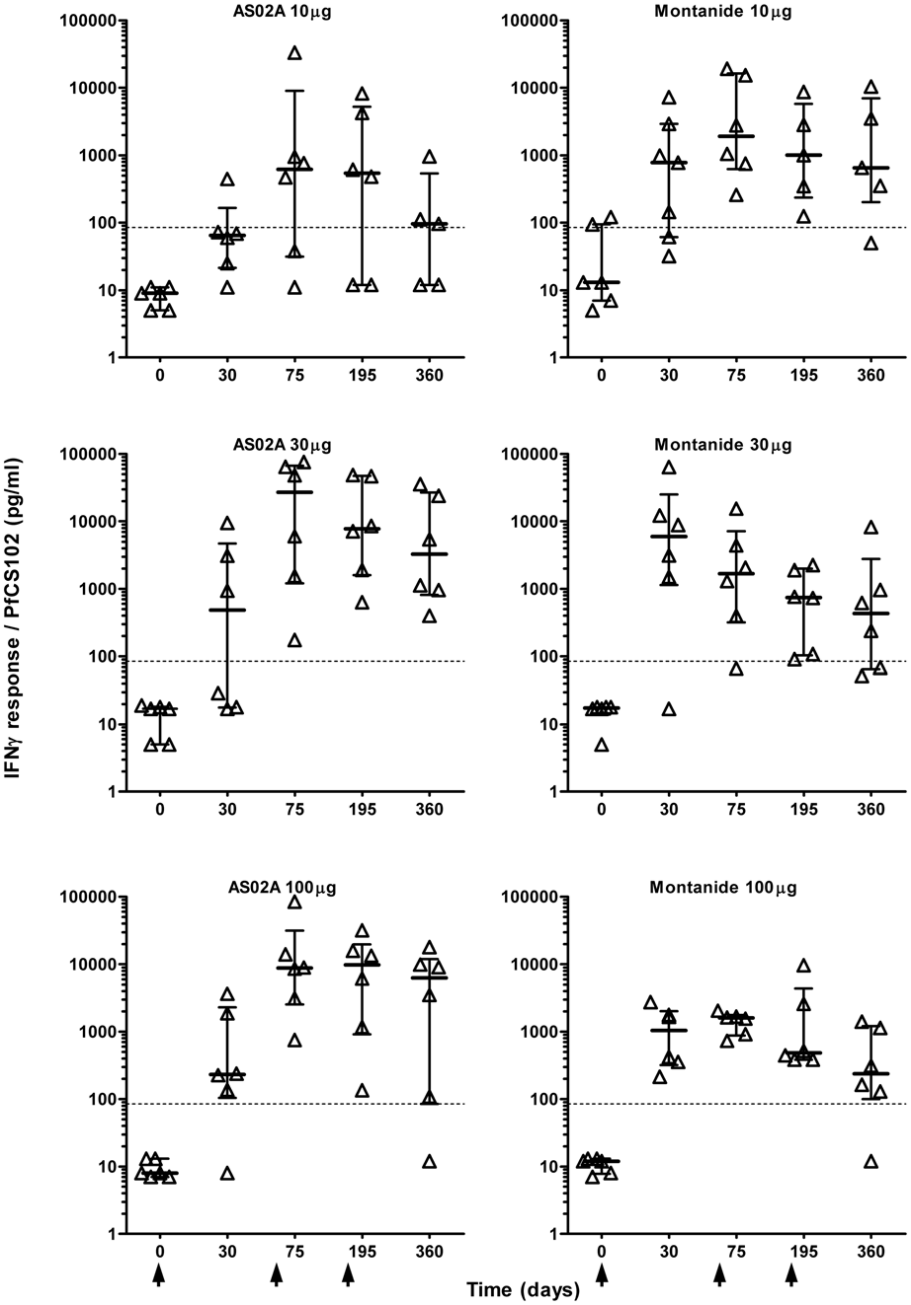
Time-course of the IFN-γ production by PBMC in response to PfCS102. IFN-γ was evaluated in the supernatant of stimulated peripheral blood mononuclear cells (PBMC). Results are expressed as individual IFN-γ secretion, expressed in pg/ml for each immunization group, with quartiles. Arrows indicate the times of vaccination.

After two immunizations, the level of the IFN-γ response in PBMC cultures (median and quartiles: 1630 pg/ml (499;8549, n = 36) increased above baseline (84.7 pg/ml) in 34 out of 36 volunteers (Figure 5). With Montanide ISA 720, significant responses were already obtained after the first injection, and no boosting effect was induced by the following injections. Moreover, there was no dose effect. With AS02A, the dose of 10 µg induced delayed responses, with only one responder after the first injection, and two non-responders overall. Whatever the dose, responses increased after the second injection, but not after the third one. Combining peptide dose groups for comparison of adjuvants, AS02A formulations induced 10-fold higher level of responses than Montanide ISA 720 after the 2^nd^ or the 3^rd^ immunization (p<0.02, generally p<0.003, Kruskal-Wallis Rank-Sum test). Like in proliferation assays, CS 310–340 and 310–383 peptides induced IFN-γ responses similar to PfCS102, CS 282–318 inducing only weak responses Overall, the frequency of volunteers responding by the production of IFN-γ (34 out of 36) was higher than in the proliferation assay (32 out of 36). However, there was a strong correlation between T cell proliferation and IFN-γ production (r = 0.8562, p<0.0001 at day 195, r = 0.8282, p<0.0001 at day 75, Spearman's rank correlation).

### IL-10 response

After immunization, the intensities of the IL-10 responses to PfCS102 increased slightly above the baseline (28.1 pg/ml) in 80% (29/36) of volunteers (median: 33.5 pg/ml, quartiles 15.8 and 77.8) after the second injection. There was no significant difference between doses or adjuvants, but there was a tendency towards higher IL-10 levels with the 100 µg dose (data not shown).

### Antigen specific CD4^+^ and CD8^+^ T cell responses

**Figure 6:**
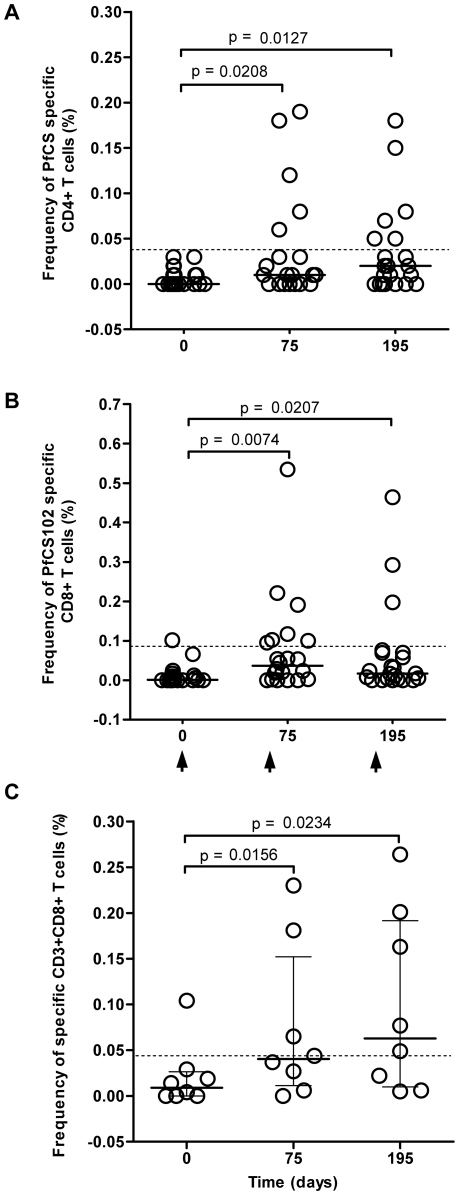
Time-course of individual frequencies of PfCS102 specific T cells. Frequencies were evaluated by intracellular cytokine staining (ICS) using the PBMC of HLA-A2 and/or HLA-A3 volunteers. Results are expressed as percentage of IFN-γ positive cells upon PfCS stimulation in the selected cell population: A, CD4^+^ cells; B, CD8^+^ cells; C, CD3^+^CD8^+^ T cells (CD16^−^/56^−^, CTL, and CD16^+^/56^+^, NKT cells) in 8 responders. Dotted lines represent baseline. Statistical analysis was based on Wilcoxon's test with *p*<0.05 as the limit of significance. Arrows indicate the times of vaccination.

The frequency of PfCS specific CD4^+^ and CD8^+^ T cells was evaluated by ICS in HLA-A2 and HLA-A3 volunteers (n = 22, 10 volunteers received AS02A, 12 received Montanide ISA 720) (Table 1). Overall, vaccination induced an increase in the frequency of malaria specific CD4^+^ and CD8^+^ lymphocytes in 16 out of 22 volunteers (73%) (Table 4). Frequencies of these responses were ranging from 0.02% to 0.19% for CD4^+^ T cells (median 0.06%) and from 0.02% to 0.53% for CD8^+^ T cells (median 0.07%). Responders with frequencies of PfCS specific CD4^+^ or CD8^+^ responses above the baseline (frequencies of 0.038% and 0.086% respectively) represented 36.2% (8/22) of volunteers (Figure 6, A and B). A further descriptive analysis of the PfCS102 specific CD8^+^ lymphocyte subsets in these 8 responders allowed to discriminate antigen specific NK (CD3^−^ CD8^+^ CD16/56^+^) from NKT cells (CD3^+^ CD8^+^ CD16/56^+^) and from CTL (CD3^+^ CD8^+^ CD16/56^−^). The 8 responders displayed a frequency of CD8^+^ NK responses that was about as high as half the frequency of IFN-γ secreting CD8^+^ lymphocytes (57.6% and 47.8% at day 75 and 195 respectively, data not shown). Nevertheless, five volunteers showed a marked increase in their antigen specific CD3^+^CD8^+^ T cells with vaccination (Figure 6C), mostly CTL (80%) and to a lesser extend NKT cells (data not shown). No distinction between doses or adjuvants could be drawn. Using the CD8^+^ cultured ELISPOT, we detected the presence of PfCS specific IFN-γ producing CD8^+^ T cells in only one volunteer out of the 14 HLA-A2 selected volunteers. This responder was one of the 5 responders by ICS described above. Virus specific CD8^+^ positive responses were obtained in 13 out of these 14 volunteers.

**Table 4:** Analysis of IFN-γ producing CD4^+^ and CD8^+^ T cell responses based on the dose of peptide per injection or the adjuvant used.

	Dose of PfCS102 (µg)			
	10	30	100	all dose levels
**CD4^+^**				
Montanide ISA 720	3/3 (100%)	2/4 (50%)	3/5 (60%)	8/12 (67%)
AS02A	2/3 (67%)	3/3 (100%)	3/4 (75%)	8/10 (80%)
Total	5/6 (83%)	5/7 (71%)	6/9 (67%)	16/22 (73%)
**CD8^+^**				
Montanide ISA 720	3/3 (100%)	3/4 (75%)	4/5 (80%)	10/12 (83%)
AS02A	2/3 (67%)	2/3 (67%)	2/4 (50%)	6/10 (60%)
Total	5/6 (83%)	5/7 (71%)	6/9 (67%)	16/22 (73%)

Values indicate numbers of volunteers presenting an individual increase in frequency of IFN-γ secreting PfCS102 specific CD4^+^ or CD8^+^ T cells, i.e. with an induced frequency above the mean + 95% confidence interval of the pre-immune specific responses in the 22 HLA-A2 and/or HLA-A3 volunteers and a delta above 0.02%.

## Discussion

### Interpretation

The present trial compared the safety and immunogenicity of PfCS102 synthetic peptide 282-383 in its GMP preparation formulated in three doses with Montanide ISA 720 or AS02A. The study demonstrated that the two adjuvant formulations were well tolerated and safe with no evidence of peptide dose effect on the occurrence of AEs (frequency and type) when administered to healthy malaria-naive adults. Formulation of PfCS102 with Montanide ISA 720 was overall better tolerated, especially locally, grading of erythema and functional limitation was definitely less in the Montanide ISA 720 group than in the AS02A group. Immunogenicity results showed that both adjuvant formulations were able to induce malaria specific responses, although the dose of 10 µg was less immunogenic in comparison to 30 µg and 100 µg and the humoral and CMI responses were more robust with AS02A. Immunizations induced marked specific anti-PfCS102 and anti-sporozoite antibody responses (35/36, at D75, 34/35 at D195) and T cell proliferative responses associated with IFN-γ production (30/36 at D75, 28/35 at D195). Specifically, 5 of 22 HLA-A2 and/or HLA-A3 volunteers developed PfCS102 specific IFN-γ secreting CD8^+^ T cell responses composed in a major part of CTL and of NKT cells. Humoral and cellular responses were only partially boosted by the third injection, and intensity plateaued at the level reached after the second injection, without substantial difference between 30 µg and 100 µg peptide doses. Moreover, these responses remained mostly stable at least for six months after the 3^rd^ injection. The candidate vaccine formulated with AS02A induced about 10-fold higher antibody and IFN-γ responses than Montanide ISA 720 formulation with doses of 30 µg or 100 µg. The safety profile and immunogenicity of the GMP PfCS102 preparation thus confirmed our initial findings with GLP PfCS102 peptide formulated in Montanide ISA 720.

Pure peptide sequences as well as highly purified proteins have poor immunostimulatory properties and need to be formulated with a potent adjuvant to induce a strong adaptive immune response. Consequently, a given antigen can clearly induce protective or non protective immunity depending on the adjuvant used, even at the same antigen dose [22]. The addition of strong adjuvants in vaccine formulations may be associated with increased reactogenicity. The direct correlation between reactogenicity and immunogenicity was well illustrated in the present study where frequency and intensity of adverse events as well as levels of immune responses were higher with AS02A than with Montanide ISA 720. The reactogenicity observed after immunisation with AS02A formulation in our study was similar to what was observed in clinical studies including prophylactic studies with malaria, tuberculosis and HIV-1 vaccines and therapeutic studies with vaccines against hepatitis B and tumour, where the vast majority of local and systemic AEs (local pain and swelling, headache, fatigue and GI) resolved within 24–48 h with no sequelae [23]. Regarding Montanide ISA 720 formulations, reactogenicity was lower in our study than previously described in a review of nine trials encompassing 296 individuals receiving Montanide ISA 720 adjuvanted vaccines by intramuscular route. Mild to moderate local reactions occurred in half the volunteers and severe local reactions in 4% of them, with rate and severity of AE appearing to be antigen and dose dependent [24]. In the current study, the reactogenicity associated with Montanide ISA 720 formulation was even lower than in our own observations with synthetic malaria peptide MSP3 injected subcutaneously [21]. This route was chosen to obtain optimal conditions for surveillance of reactivity. Simultaneously, subcutaneous injections may increase the risk of induction of large local reactions in comparison to intramuscular injections. The lower reactogenicity induced with the Montanide formulation could also be explained by the dose of adjuvant, 0.5 ml here against 1 ml in the MSP3 study, and the intrinsic character of the antigen/adjuvant mixture. At least two mechanisms, humoral via antigen specific antibodies and cellular via cytokine secreting specific T cells, may contribute to protective pre-erythrocytic immunity and concur to clear sporozoites before they reach the hepatocytes, to prevent liver invasion and to kill infected hepatocytes [25]. PfCS102 vaccine generated a strong antibody response directed against the C term of CSP and recognizing as well the native protein at the surface of the parasite. Comparison of the level of antigen specific antibody responses between different trials based on CSP vaccine is difficult in the absence of comparison of sporozoite antibody titers. The highest levels of IFAT response were generated after 3 immunizations with PfCS102 formulated with AS02A. These anti-sporozoite responses were at least equivalent to those obtained in protected individuals immunized with RTS,S/AS02A, a *Pf* CSP based malaria vaccine containing the sequence of PfCS102 [22], [26]. In these volunteers, the presence of opsonizing antibodies facilitating the uptake of sporozoites by monocytes and/or macrophages was demonstrated and proposed as a mechanism of protection [27]. Nevertheless, this protection was not associated with the recognition of a particular region of the CSP, since anti-CSP repeat and anti-C terminal antibodies where both induced. The intensity of the response to the CSP repeat, a dominant B cell epitope, was correlated with protection but probably not sufficient to induce it [28]. The protective activity of antibodies directed against the C-term of PfCS in absence of anti-CSP repeat remains to be determined.

As another mechanism, protection against natural and artificial sporozoite exposition in vaccinated individuals has been correlated with IFN-γ producing CSP specific CD4^+^ T cells [29], [30]. In this study, high IFN-γ production and proliferation by specific CD4^+^ T cells were induced as assessed in 6-day stimulation assay, particularly in AS02A groups. This Th1 response was accompanied by a low antigen specific production of IL-10, which correlated with IFN-γ production and may be related to anti-inflammatory self-regulation mechanisms. Increased frequencies of CSP specific CD8^+^ lymphocytes were also demonstrated in protected individuals after immunization with RTS/S [30]. Using the same protocol as Sun *et al.*, from 22 evaluated volunteers selected on their MHC-I, 8 clearly up-regulated their frequencies of PfCS102 specific CD8^+^ cells with vaccination. IFN-γ producing NK marker bearing CD8^+^ cells accounted for half of the total response in 5 out of these responders. Whether these cells directly or indirectly responded to PfCS peptides is not known. Nevertheless, the 5 volunteers with partial NK responses showed a low but significant increase in their frequencies of PfCS102 specific CD3^+^CD8^+^ T lymphocytes. The presence of peptide activated NK and NKT lymphocytes induced with vaccination is not *per se* prejudicial since these cells are clearly known for their abundance in the liver, for their immediate and high production of cytokines upon activation inducing the recruitment and activation of immune cells. In particular, in mice, NK cells are required at the pre-erythrocytic stage for the induction of CD8^+^ T cells necessary for protective immunity against malaria [31].

In the present study, the range of PfCS specific CD8^+^ responses was moderate and CD8^+^ T cell cultured ELISPOT did not appear sufficiently sensitive to detect responders with PfCS specific IFN-γ producing CD8^+^ T cells in comparison to ICS (1/14 and 5/22 respectively). The responses we detected were restricted to two HLA-A2 and two HLA-A3 epitopes. The use in the assay of overlapping 15-mer peptides covering all the PfCS102 sequence should have overcome the problem of genetic restriction and possibly increased the level of responses. Despite the restrictive conditions of the current analysis, we nonetheless detected 16/22 (73%) individuals with enhanced CD8^+^ specific responses, a frequency similar to that observed in our initial study, where after 3 immunizations with a GLP PfCS102, increase in CD8^+^ specific T cells was detectable in 5 out of 8 HLA-A2 volunteers [20].

In our initial study, after the 3rd vaccination with 300 µg of GLP peptide, we observed a decrease in humoral and CMI responses, which was interpreted as potential evidence of tolerization as a consequence of high antigen dose. In the present study, peptide doses were lowered to 10, 30 and 100 µg to avoid this phenomenon. Indeed, immune responses did not decrease after the 3^rd^ injection. However there was no boost effect of this 3^rd^ dose. This was despite a sufficiently large interval of 4 months between the 2^nd^ and the 3rd injections designed precisely to avoid induction of tolerance. We can thus conclude that the immune response had reached its plateau with the peptides and adjuvants used in this study. However, we cannot exclude an interest for the 3^rd^ injection in the maintenance of the immune responses at Day 360.

### Generalizibility

This Phase I study is limited in size and not powered for generalizibility to the target population. Despite this fact, the safety profile and the immunogenicity of the peptide formulations are indicative and valuable for the choice of the dose of peptide and the adjuvant for future development of a PfCS102 based vaccine as well as other peptide based vaccines.

### Overall evidence

In conclusion, PfCS102 peptide was highly immunogenic and the large array of induced immune effectors (B cells, Th1 CD4^+^ and CD8^+^ T cells, and furthermore NK cells) suggested potential for efficacy and long-term persistence of disease control. Considering that the two adjuvant formulations were well tolerated and safe, that the highest antibody and IFN-γ responses were induced with AS02A formulations, that doses of 30 µg and 100 µg of peptide induced similar responses, two or three immunizations with a dose of 30 µg of PfCS102 formulated preferentially with AS02A seem the most appropriate choice for future clinical challenge studies.

## Supporting Information

Checklist S1 CONSORT checklist
(0.12 MB PDF)

Protocol S1 Trial Protocol
(1.12 MB PDF)

Approval letter S1 Ethical Review Board approval letter
(0.34 MB PDF)

Approval letter S2 Swissmedic approval letter
(0.91 MB PDF)

Consent Form S1 Consent form
(0.06 MB PDF)
