# Supplementary material for: The Synthetic Plasmodium falciparum Circumsporozoite Peptide PfCS102 as a Malaria Vaccine Candidate: A Randomized Controlled Phase I Trial
Source: PLoS One. 2009 Oct 2;4(10):e7304. doi: 10.1371/journal.pone.0007304 (PMC2749339; doi:10.1371/journal.pone.0007304)
Supplement: Protocol S1 — Trial Protocol (1.12 MB PDF) [file pone.0007304.s002.pdf]

CONFIDENTIAL

Phase I safety and immunogenicity trial of a synthetic long peptide (282-383 or *Pf* CS 102) derived from the circumsporozoite protein of *Plasmodium falciparum,* as a candidate for an anti-malaria vaccine.

PRINCIPAL INVESTIGATOR: Dr. François SPERTINI**,** PD, MER
Division of Immunology & Allergy
Centre Hospitalier Universitaire Vaudois (CHUV)
CH - 1011 LAUSANNE
Phone: +41 21 314 07 99
Fax: +41 21 314 07 91
e-mail: Francois.Spertini@chuv.hospvd.ch

SPONSOR: Dr. Hildur E. BLYTHMAN
Director, Clinical Development
DICTAGENE S.A.
Chemin de la Vulliette 4
En Marin
CH 1014 LAUSANNE
Phone: + 41 21 785 60 20
Fax: + 41 21 785 60 21
e-mail: blythman@dictagene.ch

PROTOCOL N°: **DG001**

VERSION: Final
DATE: October 18, 2002

CONFIDENTIALITY STATEMENT

The confidential information in this document is provided to you as an Investigator, for review by you, your staff, and the applicable Institutional Review Board/Independent Ethics Committee.

Your acceptance of this document constitutes an agreement that you will not disclose the information herein to others without written authorization from RMF Dictagene SA.

CO-INVESTIGATORS:

Dr. Floriana LURATI
Centre Hospitalier Universitaire Vaudois (CHUV)
CH - 1011 LAUSANNE
Phone: +41 21 314 39 79
Fax: +41 21 314 1161
e-mail : Floriana.Lurati@chuv.hospvd.ch

Dr. Cédric Deruaz
Centre Hospitalier Universitaire Vaudois (CHUV)
CH - 1011 LAUSANNE
Phone: +41 21 314 05 13
Fax: +41 21 314 1161
e-mail : Cedric.Deruaz@chuv.hospvd.ch

Dr. Blaise GENTON, PD, MER
Policlinique Médicale Universitaire
Rue César Roux 19
CH -1005 LAUSANNE
Phone : +41 21 345 22 22
Fax : +41 21 345 2323
e-mail : Blaise.Genton@hospvd.ch
&
Swiss Tropical Institute
Socinstrasse 57
CH - 4002 BASEL
Phone : +41 61 284 82 47
Fax : +41 61 271 86 54

Biological Evaluator:

Dr. Régine AUDRAN
Division of Immunology & Allergy
Centre Hospitalier Universitaire Vaudois (CHUV)
CH - 1011 LAUSANNE
Phone: +41 21 314 08 19
Fax: +41 21 314 07 71
e-mail: raudran@ib.unil.ch

Statistician:

Dr. Thomas A. SMITH, PD
Swiss Tropical Institute
Socinstrasse 57
CH - 4002 BASEL
Phone : +41 61 284 82 73
Fax : +41 61 271 79 51
e-mail: thomas-a.smith@unibas.ch

Table of contents

PROTOCOL SYNOPSIS 4

1.0 INTRODUCTION 8

2.0 STUDY OBJECTIVES 11

3.0 STUDY DESIGN 11

4.0 STUDY POPULATION 12

5.0 STUDY PROCEDURES 14

6.0 ADVERSE EVENTS 18

7.0 EVALUATION CRITERIA 26

8.0 ANALYSIS OF THE RESULTS 27

9.0 STATISTICS 28

10.0 STUDY MATERIAL 30

11.0 STUDY AMENDMENT / TERMINATION 31

12.0 ADMINISTRATIVE ASPECTS 32

13.0 REGULATORY AND ETHICAL CONSIDERATIONS . . . 34

14.0 INVESTIGATORS RESPONSIBILITIES 36

15.0 SIGNATURE SHEET 37

ANNEXES

A. References 38

B. Abbreviations 42

C. Methods for the preparation of the vaccines for injection 43

D. Definitions relating to Adverse Events 46

E. Sample of the Informed Consent Form 49

F. Declaration of Helsinki 55

PROTOCOL SYNOPSIS

Title:	**Phase I safety and immunogenicity trial of a synthetic long peptide (P*f* 282-383 or *Pf CS* 102), derived from the circumsporozoite protein of *Plasmodium falciparum,* as a candidate for an anti-malaria vaccine.**
Protocol version:	Final (18.10.2002)
***Name of the product*:**	**P*f* CS 102** (P*f* CS 282-393)
***Adjuvants compared*:**	Montanide ISA 720 (SEPPIC)<br>AS02A (GlaxoSmithKline)
***Route of administration*:**	Intramuscular injection into the deltoid muscle.
Schedule:	Three injections, at weeks 0, 8 and 26.
***Study design*:**	Single centre, open (adjuvant-blinded) block randomized, comparative study of 2 different adjuvants, each tested with 3 escalating doses of antigen.
Study objectives:	1. to demonstrate the safety and the tolerability of the administration of the synthetic peptide P*f* CS 102, given in combination with different adjuvant molecules, as a potential anti-malaria vaccine.<br><br>2. to determine the optimal antigen/adjuvant combination which will induce the best humoral + cellular immune response.
***Number of subjects*:**	36 evaluable subjects.
Subject population:	Adult normal volunteers.
Randomization:	Six subjects will be enrolled per antigen dose and per adjuvant, for a total of 36 subjects. Subjects (3 males and 3 females) will be randomized for each vaccine (antigen/adjuvant) dose, starting with 10 µg Pf CS 102, followed, at least 3 weeks later, with 30 µg Pf CS 102, and again, at least 3 weeks later, with 100 µg Pf CS 102.

Study procedures: Volunteers will be screened, enrolled and followed by the co-investigator, Dr. Lurati, under the responsibility of the principal investigator, Dr. Spertini, at the Vaccinology center of the Division of Immunology and Allergy, CHUV, Lausanne.

<u>Schedule of injections for each group</u>

**Visit N°**	**1**	**2**	**3**	**4**	**5**	**6**	**7**	**8**	**9**	**10**	**11**	**12**
Week	***-1***	***0***	***0***	***4***	***8***	***8***	***10***	***26***	***26***	***28***	***52***	***78***
**Timelines (days)**	- 7	0	2	30	60	62	75	180	182	195	360	540
**Windows (days)**	±7			±4	±4			±4		±4	±7	±14
**Physical examination**	X	X	X		X	X		X	X		X	X
**Vaccinations**		**Inj. 1**			**Inj. 2**			**Inj. 3**				

<u>Timepoints for safety – tolerability tests and immunological assessments</u>

**Visit N°**	**1**	**2**	**3**	**4**	**5**	**6**	**7**	**8**	**9**	**10**	**11**	**12**
Week	***-1***	***0***	***0***	***4***	***8***	***8***	***10***	***26***	***26***	***28***	***52***	***78***
**Timelines (days)**	- 7	0	2	30	60	62	75	180	182	195	360	540
**Windows (days)**	±7			±4	±4			±4		±4	±7	±14
**Safety & Tolerability**	X			X			X			X	X	
**Immunology tests**	X	X		X	X		X	X		X	X	

Surveillance of AEs and follow-up

Solicited symptoms are adverse events reported by the subjects, which occur immediately after the injection, or any time between the injection and the 4-day follow-up period.

Unsolicited symptoms are all adverse events reported by the subjects which begin **after** the 4 day follow-up period for solicited symptoms. Space on the diary cards and CRF will be allocated for the recording of unsolicited symptoms. Every effort should be made by the investigator to explain each adverse event and assess its causal relationship, if any, to administration of the study vaccine(s); all data must be transcribed into the CRF.

***Study endpoints*:**

Safety endpoint: Occurrence and severity of "solicited" adverse events (according to a standard list) and "unsolicited" AEs, during the procedure, the 6-month follow-up (interim analysis) and the 18-months follow-up period (final endpoint).

Immunogenicity endpoints: antibody response (ELISA) (IFA); cellular response (CD4+ T-cell proliferation and IFN-γ production, CD8+ IFN-γ production) (ELISpot).

Statistical analysis and presentation of results:

All patients who received at least one dose of study vaccine will be included in the *safety population.* Safety data analyses will be performed on the safety population.

Subjects will be included in the final analysis of *immunogenicity* if they fulfill the following criteria:

- all inclusion/exclusion criteria were respected
- they received the vaccine they were randomised to receive
- they received a second dose of the vaccine
- allowed intervals between injections were respected
- allowed intervals between blood sampling schedules were respected
- no forbidden vaccine or concomitant medication were taken

All data will be reported in subject listings.

Safety data: Data will be collected at baseline (V1) and at 4, 10, 28 and 52 weeks will be listed for the study group.

Adverse Events: Descriptive statistics will be used to analyse adverse events (AEs), including intercurrent illnesses, for the study group. The number of AEs and their severity will be assessed. Safety data will be reported using frequency tables of numbers of AEs. With

frequently occurring (10 or more) event types, effects of adjuvant and vaccine dose will be tested using logistic regression models, with both the type of adjuvant and vaccine dose as factors in the model. Statistical significance will be assessed by likelihood ratio tests.

Immunogenicity Data: Immunological data for each time point will be analysed separately.

Descriptive statistics of antibody levels determined by the specific anti-P*f* CS 102 antibody titers at baseline (V2) and at 4, 8, 10, 26, 28 and 52 weeks.

Descriptive statistics of lymphocyte stimulation indices, as determined by the proliferative $CD4^+$ T-cell and/or the $CD8^+$ IFN-γ producing T-cell response.

Assessment of new responders for humoral and cell-mediated immunity after each immunisation and overall.

****

1.0 INTRODUCTION

1.1 Background information

The repeated exposure of man to malaria parasites generates an immune response which leads to a certain degree of protection, so that the subject is less prone to becoming sick, but a complete protection is never obtained. However, a complete protection against repeated exposure to living sporozoites was achieved by using irradiated sporozoites as vaccination material [1 - 3]. Studies performed in animals and in human volunteers have demonstrated that the anti-sporozoite protection depends on both a humoral and a cellular immune response [4 - 8].

In the protection obtained with irradiated sporozoites, one protein, the "circumsporozoite" (CS), abundantly found on the surface membrane, plays a crucial role in the induction of the immune response. This immune response is related to different epitopes: the B epitope [9], T helper [6] and T cytotoxic [10 - 14]. Attempts to establish a protective immune response based on the sole utilization of a dominant B epitope, found on the central domain of the CS protein, were not satisfactory [15 – 16], partly due to a low immunogenicity of this type of preparation. It has been demonstrated that this low immunogenicity could be enhanced by adding T helper epitopes [17 – 18] obtained from exogenic epitopes, such as the "universal epitope from tetanus toxin [19] or epitopes derived from CS itself [20 - 21]. A protection could also be obtained in mice by the transfer of cytotoxic CD8 T cells [13] [22] The role of cytotoxic T cells in the control of malaria has been well documented [23]. These observations lead to the combined utilization of B, T helper and T cytotoxic epitopes, mimicking the immune response obtained with irradiated sporozoites. The use of long fragments of the CS protein provides a wide range of epitopes, capable of being recognized at the same time by the HLA Class I and HLA Class II of the subject, without having to define the individual HLA restriction prior to the immunization. [24 – 27].

Different CS protein sequences, obtained as recombinant products, have been tested as vaccines, and a certain degree of protection has been observed [28 – 30]. One approach has been to use long synthetic peptides, which have the advantage of obtaining, rather rapidly, clinical grade material of high degree of purity (> 90%) and extremely low intrinsic toxicity. In animal tests, long synthetic peptides, coding for the C terminal region of the CS protein 282-383 (P*f* CS 102) were found to be highly immunogenic in mice, generating a T helper and T cytotoxic response, as well as a humoral response able to block the intra-hepatocyte passage of the sporozoites of *Plasmodium falciparum* [31 – 32]. In addition, it was found that individuals living in malaria endemic regions of South America and Africa have high titers of specific anti-P*f* CS 282-383 antibodies. It has also been possible to demonstrate a significant *in vitro* T cellular immune response with peripheral lymphocytes from human donors, from endemic and non-endemic regions [33]. In a pre-clinical phase, the CS protein was tested in a South-American monkey, *Aotus lemurinus,* and it was found to be extremely immunogenic [34].

Recently, a Phase I study, in human healthy volunteers, was completed using P*f* CS 282-

383 produced under GLP conditions; two doses, 100 µg and 300 µg, combined with 2 adjuvants, Aluminum hydroxide and Montanide ISA 720, repeated 3 times (at 0, 1 and 6 months) were compared [35]. Results were very encouraging, as no severe side-effect was observed, and only one subject presented a mild local reaction (erythema and slight pain during the first 48 hours following the injections). The humoral immune response induced with Montanide was excellent (8/8 subjects); the cellular immune response (T-cell proliferation and $CD8^+$ γIFN producing T-cells) was also satisfactory.

The potential risks involved following the injection of synthetic long peptides has already been studied in preliminary studies, and no serious adverse reaction was observed. The adjuvants used in this trial have already been extensively used by others) and did not present any short-term or long-term “toxic” effects. However, Montanide ISA 720 led to severe AEs with other malarial antigens (recombinant proteins) [38] [40 - 41] or HIV antigen [39] but usually only with high doses.

More than 1000 people have been vaccinated with formulations containing Montanide ISA 51, which is a mineral oil based emulsion, and various publications report a good tolerance of this adjuvant. Montanide ISA 720, based on non-mineral oil, is also in current human clinical trials, and phase I and II demonstrate the good tolerance of the product [36 – 37]. The most common effects described were fever, local pain and tenderness; in a few cases, an induration and erythema were also recorded in the placebo group, whereas a number of granulomas and a few sterile abcesses were observed in the vaccine group, and were antigen-dose dependent [39].

The clinical safety of AS02A-containing vaccines has been assessed in 30 clinical studies comprising prophylactic studies with malaria and HIV-1 vaccines and therapeutic studies with hepatitis B, genital warts, herpes and tumour vaccines. Up to August, 2000, a total of at least 1298 subjects (age range 15-84 years) received 3938 doses of AS02A-containing vaccines. The most common local symptom across all studies was injection site pain, sometimes associated with redness, swelling, and/or a transient local muscle soreness and stiffness. None of the reactions required medication, and generally resolved within one week after the injection. The nature and pattern of the symptoms were generally mild to moderate in intensity and resolved without sequelae. Systemic symptoms observed included headache, malaise/fatigue, fever/flu-like symptoms, nausea, vomiting and myalgia. Nearly all grade 3 symptoms (defined as those that prevent normal everyday activities) probably or suspected to be related to vaccination resolved within 3-7 days. Only three serious adverse events were suspected of being causally related to vaccination with a AS02A-containing vaccine. One subject developed jaundice and a transient increase in serum alanine aminotransferase. The subject was semi-immune to malaria and a chronic carrier of the hepatitis B virus. All symptoms resolved within approximately 2 weeks without sequelae. Two further subjects, who received the genital warts vaccine, reported flu-like symptoms which resolved within 2-3 days without sequelae. No other clinically significant haematological or biochemical abnormalities have been observed. AS02A-containing vaccines did not elicit any serious adverse events that necessitated their immediate withdrawal from clinical trials. The AS02A adjuvant has been shown to be safe, as reflected in the low incidence of both vaccine-related serious and non-serious adverse events, with no

significant increase in reactogenicity following multiple doses.

1.2 Study rationale

In view of the results obtained with the preliminary Phase I trial using the long P*f* CS 282-383 synthetic peptide (P*f* CS 102), and in view of the intention to obtain a future marketing approval for the use of this peptide as a vaccine, a batch of the same peptide, has been prepared under GMP conditions. In addition, certain production conditions have been modified (the oxidizing procedure of the cysteines present in the peptide structure). Moreover, it is planned to compare the immunogenicity obtained with Montanide ISA 720 with the immune response induced by another adjuvant, AS02A (GlaxoSmithKline Biologicals. [30].

This trial should also confirm the previous observation concerning the safety and tolerability of the administration of P*f* CS 102. Moreover, the immunogenicity obtained with Montanide ISA 720 suggests that an anti-malaria vaccine based on the use of the CS protein should provide protection against infection with the parasite; this would justify implementing the demonstration of this protection by exposing immunized subjects to the experimental infection with living parasites, in a follow-up Phase IIa trial. The CS 102 / adjuvant combination giving the best results in this Phase I trial would be chosen for the planned Phase II study.

****

2.0 STUDY OBJECTIVES

The objectives of this trial are twofold:

1. to demonstrate the safety and tolerability of the intra-muscular administration of the synthetic long peptide P*f* CS 102, given in combination with 2 different adjuvant molecules, as a potential anti-malaria vaccine.

2. to determine the optimal antigen-adjuvant combination, i.e. the combination inducing the best humoral + cellular immune response (best anti-P*f* CS antibodies + best cytotoxic activity) by measuring:

a) the specific anti-P*f* CS 102 antibody response;
b) the CD4+ T-cell proliferation and IFN-γ production in response to P*f* CS 102 peptides;
c) the CD8+ T-cell proliferation and IFN-γ production in response to P*f* CS 102 peptides;
d) the recognition of the parasite in a direct immuno-fluorescence test.

3.0 STUDY DESIGN

3.1 Overall design

This prospective, randomized, comparative study will be conducted in healthy normal volunteers. Subjects will be enrolled into six groups: 10 µg P*f* CS 102 with either Montanide ISA 720 or AS02A; 30 µg P*f* CS 102 with either Montanide ISA 720 or AS02A; 100 µg P*f* CS 102 with either Montanide ISA 720 or AS02A. There will be 6 subjects per group.

All subjects will be evaluated before the first injection (baseline), and after each injection. The examinations to be done at each of these evaluations are described in chapter 5.0. An interim analysis will be performed with data collected at 10 weeks (V7 = d75); a final analysis will be performed after the 12-month follow-up data has been collected for all subjects enrolled in the trial. (c.f.: 9.4).

3.2 Study duration

<u>Duration of inclusion period:</u> two months.

<u>Duration of follow-up per subject:</u> Each subject enrolled in this study will be followed 18 months after treatment.

<u>Total planned duration:</u> ~ 20 months.

4.0 STUDY POPULATION

4.1 Patient enrollment

A total of 36 subjects will be enrolled in the study. Each subject complying with the inclusion / exclusion criteria will be randomized to one of the 6 antigen/adjuvant groups, as indicated below, in accordance with the randomization envelopes. Each antigen/adjuvant group will consist of 6 subjects (3 males and 3 females).

4.2 Inclusion criteria

1. Volunteers of both sexes, aged between 18 and 45 years.
2. Subjects who have undergone a thorough clinical checkup and are in good health.
3. Subjects who have understood the purpose of the study, have signed the informed consent, will be available and are reachable by telephone throughout the study duration.
4. For female volunteers, a negative pregnancy test.
5. For female volunteers, an adequate contraception throughout the study duration.

4.3 Exclusion criteria

The following criteria should be checked at the time of study entry. If any apply at the time of study entry, the subject must not be included in the study:

1. Known history of previous contact with *Plasmodium sp* or recent travel (within the last 3 months) or long-term residence in a malaria endemic region, or previous vaccination with an experimental malaria vaccine.
2. Positive anti-P*f* CS 102 peptide immune response, measured by ELISA.
3. Known history of hypersensitivity to any component of the vaccine (MPL or QS21) or previous vaccination with a vaccine containing MPL or QS21.
4. Volunteers are not allowed to receive any other vaccine during the 3 months preceding the 1st injection and until after 3 months following the 3rd injection. If the subject needs to be vaccinated during this period, he/she will be withdrawn from the study.
5. Chronic administration (defined as more than 14 days) of immunosuppressants or other immune-modifying drugs within six months prior to the first vaccine dose. (For corticosteroids, this will mean prednisone, or equivalent, ≥ 0.5 mg/kg/day. Inhaled and topical steroids are allowed.)
6. Family history of congenital or hereditary immunodeficiency.
7. Acute disease at the time of enrolment. '*Acute disease' is defined as the presence of a moderate or severe illness with or without fever. All vaccines can be administered to persons with a minor illness such as diarrhea, mild upper respiratory infection without or with low-grade temperature, i.e: axillary temperature (≤37.5°C).*

8. Acute or chronic, clinically significant pulmonary, cardiovascular, hepatic or renal functional abnormality, as determined by physical examination or laboratory screening tests.
9. Any laboratory data collected during the pre-screening phase found to be outside the normal ranges defined by the CHUV Central Laboratories (except for the values for GOT, GPT and creatinine, where values 20 % above the normal range will be admitted). Positive tests for HIV, HBV or HCV (indicating active hepatitis).
10. Pregnant or breast-feeding women.
11. History of drug or psychotropic drug abuse (which interferes with a normal lifestyle) during the previous year.
12. Volunteers for which follow-up is compromised due to socio-cultural, geographic or psychological reasons.
13. Any other significant finding that in the opinion of the investigator would increase the risk of having an adverse outcome from participating in the study.
14. Volunteers already participating in another clinical trial.
15. Volunteers should not plan to travel to a malaria endemic zone during the 12 months following their enrolment into the study.

NB: Volunteers must not perform any physical exercise within the span of 4 hours before and after receiving a vaccine injection, and cannot donate their blood during the whole duration of the trial.

For statistical purposes:

1. If a subject *does not agree to inclusion* (does not sign the consent form), he/she will not participate. (If the investigator has started to complete a CRF (Case Report Form) for such a subject, data in this CRF will not be taken into account in any analysis, even if collected by the sponsor).

2. If a subject signs the consent form but *withdraws his/her consent before injection*, he/she will not participate. If the investigator has started to complete a CRF for such a subject, data in this CRF will not be taken into account in any analysis, even if collected by the sponsor.

3. If a subject signs the consent form but *withdraws his/her consent after the 1st injection*, he/she will abandon the study as of the date of withdrawal. The subject will attest the withdrawal on the consent form and the reason shall be noted at the end of the CRF. Data collected until the date of withdrawal will be used for the statistical analysis of safety.

4. Finally, if a subject is *lost to follow-up*, this fact shall be noted at the end of the CRF. A subject will be considered as lost to follow-up if he/she does not come back to 2 consecutive follow-up visits, even after repeated telephone calls or letters by the investigator. Data collected for such subjects will be used for the statistical analysis until the date of lost to follow-up.

5.0 STUDY PROCEDURES

The study will be performed at the Vaccinology centre of the Division of Immunology and Allergy, CHUV, Lausanne. Volunteers will be enrolled and followed by the co-investigator, Dr. Lurati, under the responsibility of the principal investigator, Dr. Spertini. Dr. Lurati will be responsible for the transfer of the blood samples to the CHUV diagnostics laboratories, where they will be processed by Dr. Audran, for the immunologic parameters.

5.1 Baseline assessment (Visit 1: pre-screening: days -15 to –1)

Candidates for this study must meet all of the inclusion criteria and will be excluded if any of the exclusion criteria conditions apply. Subjects who are judged eligible by the investigator, will sign the Informed Consent form, after the Investigator has explained the purpose of the study. Demographic data will be collected, and a complete clinical history will be recorded, and laboratory tests (including HIV, HBV and HCV) will be performed. In the event that a subject is tested positive for HIV, he/she will be informed and referred for counseling and treatment.

5.2 Randomization

Subjects will be stratified in subgroups, according to the following combinations:

Antigen / Adjuvant	P*f* CS 102 10 µg	P*f* CS 102 30 µg	P*f* CS 102 100 µg
Montanide ISA 720	I	III	V
AS02	II	IV	IV

For each antigen dose, subjects will be randomized by blocks of 6, 3 to each adjuvant. There will be two blocks for each antigen dose, one comprising male subjects and one females. The Statistician will prepare the randomization envelopes before the start of the study and provide one set to the sponsor and one set for the Data Management contractor.

The first volunteers should be enrolled in November 2002. The groups receiving 10 µg of the combination P*f* CS 102/adjuvant (I & II) will be injected first; the groups receiving 30 µg (III & IV) will be injected at least three weeks later, to allow for the evaluation of any adverse effect to the vaccine; the last groups receiving 100 µg (V & VI) will be injected at least three weeks later.

5.3 Vaccination of volunteers:

Blood samples will be collected when required by the protocol. One fraction of blood samples will be transported to the central laboratory of the CHUV (Haematology and Chemistry); the other fraction will be transported to the Division of Immunology at the CHUV. A form with exact address, shipping time and receipt time will be joined to the blood samples.

Individual vaccine doses will be prepared as described in Annex C. The vaccine will be given by an intra-muscular injection into the deltoid muscle. The second and the third injections will be done on alternating sides: i.e. [left > right > left] or [right > left > right], unless there is a justified reason for not doing so.

The vaccines will be administered under the supervision of physicians skilled in the management of anaphylactic reactions. The vaccinees will be observed closely for at least 1 hour, with appropriate medical treatment readily available in case of a rare anaphylactic reaction following the administration of the vaccine.

5.4 Assessment at the follow-up visits (Visit 2 to Visit 12)

There will be 12 visits scheduled, as indicated on the flowchart (cf. 5.4.1). Volunteers will receive 3 intra-muscular injections of the P*f* CS 102 peptide antigen combined with the different adjuvants (Montanide ISA 720 or AS02A). In women of childbearing age, a pregnancy test will be performed before each injection. A complete physical examination, including urinalysis, will be performed on the days indicated in the flowchart (cf. 5.4.1). Other clinical checkups may be performed, if judged necessary.

Subjects will be followed during the first hour following the injection. They will report again to the site 48 hs. later, to submit to reactogenicity evaluations and to have their temperature measured. At each visit, any adverse event will be recorded in the subject's Case Report Form (CRF) book. Subjects will note any signs or symptoms they notice following the injections, on a Diary Card provided to them. Body temperature (axillary) should be recorded daily, in the evening, from the day of injection and the following 4 days.

Eight blood samples will be taken, to perform the biological and immunological tests. Labels will show only the randomization number; samples will be transferred immediately to the CHUV. Plasma samples will be kept at – 80°C. Cellular tests will be performed immediately, with fresh cells. (Cell aliquots will be kept for future reference).

Any medication that the volunteers may be taking before inclusion, or at any time during the trial, must be recorded in the "Concomitant medication" pages of the CRF book.

5.4.1 Flowchart of study procedure

**Visit No.**<br>***Week***	**V1**<br>***-1***	**V2**<br>***0***	**V3**<br>***0***	**V4**<br>***4***	**V5**<br>***8***	**V6**<br>***8***	**V7**<br>***10***	**V8**<br>***26***	**V9**<br>***26***	**V10**<br>***28***	**V11**<br>***52***	**V12**<br>***78***
**Timelines (days)**	- 7	0	2	30	60	62	75	180	182	195	360	540
**Windows (days)**	±7			±4	±4			±4		±4	±7	±14
**Vaccinations**		**1**			**2**			**3**				
**Physical examination**	*	•	•		*	•		*	•		•	*
**(1) Safety & tolerability**	•			•			•			•	•	
**Blood samples**	•	•		•	•		•	•		•	•	
**(2) ELISA anti-CS102**	•	•		•	•		•	•		•	•	
**(3) IFA anti-parasite**		•								•		
**(4) CD4 anti-CS102**		•		•			•			•	•	
**(5) CD4 anti-parasite**										•		
**(6) CD4 anti-peptides**										•		
**(7) CD8 ELISPOT**		•					•			•		
**(8) CD8 tetramer staining**		•					•			•		
**(9) Other T-cell responses**		•					•			•		

(*) Complete physical examination

(1) <u>Laboratory tests</u>:
- Full haemogramme (RBC, haematocrit, Hb, platelets, WBC) [+ HLA-A*0201 typing on V1]
- MCV – MCH – MCHC
- ASAT, ALAT, total bilirubin, alkaline phosphatase, gamma GT, creatinine.
- HIV, HCV and HBV (screening test) at V1 (day 7 ±7)
- For women, urine pregnancy test before each vaccine injection.

<u>Immunology tests</u>:
(2) Anti- P*f* CS 102 antibodies (ELISA).
(3) Anti- parasite antibodies (IFA)
(4) Anti- P*f* CS102 CD4 T-cell proliferation and γ–INF production

(5) Anti- parasite CD4 T-cell proliferation and γ–INF production
(6) Anti- P*f* CS 327-335 and P*f* CS 299-308 CD4 T-cell proliferation and γ–INF production
(7) γ–INF production by $CD8^+$ T-cells in HLA-A*0201 subjects
(8) The analysis of the $CD8^+$ tetramer staining is a complementary test being developed.
(9) The analysis of the other T-cell responses (CD8, CD45-RA, CD27, CD69, CD25-IL2) is a complementary test being developed.

NB: Results of tests (8) and (9) will not be used as Go / No Go decision criteria.

5.5 Subject withdrawals / dropouts / lost to follow-up

All subjects who received at least one dose of study vaccine: i.e.: *safety population,* will be included in the safety data analyses.

Subjects may be withdrawn from the *immunogenicity population* analyses if the following conditions are verified:

1. Any inclusion/exclusion criteria were not fulfilled.
2. A subject received a different peptide/adjuvant mixture from the one they were randomised to receive.
3. A subject received only the 1st injection of the vaccine ("early withdrawal").
4. A subject who does not report for the scheduled visits within the windows indicated in the calendar (windows range between 2 and 14 days, depending on the visit schedule).
5. Any subject whose blood samplings were not collected according to the timetable above.
6. Any subject found to be taking any medication having an immunosuppressive or immuno-modulator effect, or taking any vaccination during the first 7 months of the trial.

An "early" or "premature" withdrawal is one taking place before the 2nd injection. NB: Any early withdrawal will be replaced.

In addition, subjects will be withdrawn from receiving further vaccinations in the following situations:

1. A subject presents an induration at the site of the injection, following the 1st or 2nd injection, measuring ≥12 cm in diameter: the 2nd or 3rd injection will not be given, but the subject will be followed for the duration of the trial.
2. A serious adverse event, which requires withdrawal from the trial, is observed at any time during the duration of the trial (cf. chapter 6.0 and Annex D).
3. A subject becomes pregnant during the study period.
4. A subject freely chooses to withdraw from the trial.
5. Death.

6.0 ADVERSE EVENTS

The recording of adverse events is an important aspect of study documentation. It is the responsibility of the investigator to document all adverse events according to the detailed guidelines set out below.

The subjects will be instructed to contact the investigator immediately should they manifest any signs or symptoms they perceive as severe.

6.1 Definitions (see also Annex D)

Adverse event (AE): An adverse event includes any noxious, pathological or unintended change in anatomical, physiological or metabolic functions as indicated by physical signs, symptoms and/or laboratory detected changes occurring in any phase of the clinical study, whether or not considered vaccination related. This includes an exacerbation of pre-existing conditions or events, inter-current illnesses, or vaccine or drug interaction.

Anticipated day-to-day fluctuations of pre-existing conditions, that do not represent a clinically significant exacerbation need not be considered adverse events. Discrete episodes of chronic conditions occurring during a study period should be reported as adverse events in order to assess changes in frequency or severity.

Adverse events should be documented in terms of a medical diagnosis. When this is not possible, the adverse event should be documented in terms of signs and symptoms observed by the investigator or reported by the subject at each study visit.

Adverse events which occur after informed consent is obtained, but prior to vaccination, will be documented in the Medical History form within the subject's CRF.

Serious Adverse event (SAE): A serious adverse event is any untoward medical occurrence that:

- results in death or
- is life threatening or
- results in persistent or significant disability/incapacity or
- requires in-patient hospitalization or prolongation of existing hospitalization or
- is a congenital anomaly/birth defect in the offspring of a study subject.

In addition, important medical events that may jeopardize the patient or may require intervention to prevent one of the other outcomes listed above should be considered as serious. (Examples of such treatments are intensive treatment in an emergency room or at home for allergic bronchospasm; blood dyscrasias or convulsions that do not result in hospitalization; or development of drug dependency or drug abuse.)

Hospitalization for either elective surgery related to a pre-existing condition which did not increase in severity or frequency following initiation of the study, or for routine clinical procedures (including hospitalisation for "social" reasons) that are not the result of an adverse event, must be recorded in the CRF. If the hospitalisation arises from a pre-existing condition, or was planned prior to the first vaccination, it should be recorded in the Medical History form of the CRF. If it was planned after the first vaccination, it should be recorded in the AE page of the CRF. In both cases, it should be recorded as 'Hospitalisation (Not an adverse event)', and the relationship to vaccination will be checked "No".

Although not considered as a 'serious adverse event', cancer should be reported among the serious adverse events.

6.2 Recording of expected and unexpected adverse events

At each visit, all adverse events either observed by the investigator or one of his clinical collaborators or reported by the subject, spontaneously or in response to a direct question, will be evaluated by the investigator. Adverse events not previously documented in the study will be recorded in the Adverse Event form within the subject's CRF. The nature of each event, date and time (where appropriate) of onset, outcome, intensity and relationship to vaccination should be established. Details of any corrective treatment should be recorded on the appropriate page of the CRF.

6.2.1 <u>Solicited adverse events</u>

Solicited symptoms are adverse events specifically asked for by the Investigator from the subjects, according to the table below, which occur immediately after the injection, or any time between the injection and the 4-day follow-up period.

**Local (at the injection site)**	
	1. Pain
	2. Erythema
	3. Itching
	4. Induration
	5. Limitation of arm motion

N.B.: Solicited local adverse events are considered "vaccine-related".

General / Systemic	
	1. Erythema (generalized)<br>2. Exanthema<br>3. Urticaria<br>4. Fatigue<br>5. Fever (Axillary temperature)<br>6. Joint pain<br>7. Myalgia<br>8. Headache<br>9. Dyspnea<br>10. Fainting<br>11. Dizziness<br>12. Sweating<br>13. Nausea<br>14. Vomiting<br>15. Diarrhea<br>16. Palpitations

N.B.: Temperature will be recorded in the evening. Should temperature measurement additionally be performed at another time of day, the highest temperature will be recorded.

6.2.2 Unsolicited adverse events

Unsolicited symptoms are any symptoms or adverse events recorded between vaccination and 30 days after vaccination, which begin after the 4 day follow-up period for solicited symptoms. It also covers all core symptoms (= solicited) starting after the 4-days post-vaccination period until 30-days post-vaccination. Space on the diary cards and CRF will be allocated for the recording of unsolicited symptoms.

6.3 Assessment of intensity

For the classification of the intensity of local adverse events, maximum intensity will be assigned as follows:

Adverse event	Grade	Intensity
Pain	0	Absent
	1	Painful on touch
	2	Painful when limb is moved
	3	Prevents normal activity
Erythema		Record greatest surface diameter in mm

	0	No erythema
	1	> 0 - 20 mm
	2	> 20 - 50 mm
	3	> 50 mm
Itching	0	Absent
	1	Symptom which is easily tolerated
	2	Symptom that interferes with normal activity
	3	Symptom that prevents normal activity
Induration		Record greatest surface diameter in mm
	0	0 mm (No induration)
	1	> 0 - 20 mm
	2	> 20 - 50 mm
	3	> 50 mm
Limitation of arm motion - Abduction at the shoulder	0	None
	1	>90° but <120°
	2	>30° but ≤ 90°
	3	≤ 30°

Intensity of the <u>general</u> adverse events should be assessed as described below:

Adverse event	Grade	Intensity
Fever		Record axillary temperature in °C
	0	< 37.5°C
	1	37.5 ≤ T° < 38.0°C
	2	38.0 ≤ T° < 39.0°C
	3	≥ 39.0°C
Other adverse events	0	No adverse event
	1	An adverse event which is easily tolerated by the subject, causing minimal discomfort and not interfering with everyday activities.
	2	An adverse event which is sufficiently discomforting to interfere with normal everyday activities.
	3	An adverse event which prevents normal, everyday activities, i.e. prevents attendance at work and necessitates the administration of corrective therapy

For all other adverse events, maximum intensity should be classified following the same criteria listed above.

6.4 Assessment of causality

Every effort should be made by the investigator to explain each adverse event and assess its causal relationship, if any, to administration of the study vaccine(s).

The degree of certainty with which an adverse event can be attributed to administration of the study vaccine(s) (or alternative causes, e.g. natural history of an underlying disease, concomitant therapy, etc.) will be determined by how well the event can be understood in terms of one or more of the following:

- Reaction of similar nature having previously been observed with this type of vaccine and/or formulation.
- The event having often been reported in the literature for similar types of vaccines.
- The event being temporally associated with vaccination or reproduced on re-vaccination.

Causality of all other adverse events should be assessed by the investigator using the following method: did the vaccine possibly contribute to the adverse event?

NO:	The adverse event is not causally related to administration of the study vaccine(s). There are other, more likely causes and administration of the study vaccine(s) is not suspected to have contributed to the adverse event.
YES:	There is a reasonable possibility that the vaccine contributed to the adverse event.

6.5 Follow-up of adverse events and assessment of outcome

Investigators should follow subjects with serious adverse events until the event has resolved, subsided, stabilized, disappeared, the event is otherwise explained, or the subject/patient is lost to follow-up; or, in the case of non-serious adverse events, the subject/patient completes the study.

Clinically significant laboratory abnormalities, as well as any adverse event, will be followed up until they have returned to normal, or a satisfactory explanation has been provided. Reports relative to the subsequent course of an adverse event noted for any subject must be submitted to the Study Monitor.

Outcome should be assessed as:

1 = Recovered
2 = Recovered with sequelae
3 = Ongoing at subject study conclusion (active phase)

4 = Died
5 = Unknown

6.6 Reporting

Adverse Events (AE) All adverse events occurring within one month (minimum 30 days) following administration of each dose of vaccine must be recorded on the Adverse Event form in the subject's CRF, irrespective of severity or whether or not they are considered vaccination-related. A phone call will be made by the Investigator on day 30 following the 2nd and 3rd injection, to have an update on the AE's status: to obtain an update on previously ongoing AEs and to collect information on any new AEs starting within the period of follow-up for unsolicited AEs. Subjects will be requested to come to the site for unresolved AEs related to vaccination.

At each visit, all adverse events, either observed by the investigator or one of his clinical collaborators or reported by the subject spontaneously or in response to a direct question will be evaluated by the investigator. Adverse events not previously documented in the study will be recorded in the AE form within the subject's CRF. The nature of each event, date and time (where appropriate) of onset, outcome, intensity and relationship to vaccination should be established. Details of any corrective treatment should be recorded on the appropriate page of the CRF.

Serious Adverse Events (SAE) The Investigator will report any SAE to the sponsor as soon as possible, but not later than 1 working day after the event occurred. All SAEs should be documented on the Serious Adverse Event Section in the Case Report Form, along with an explanation of any medical treatment administered. The form must be completed, signed and sent by fax to the sponsor study monitor. All SAEs occurring in the period between the 1st vaccine dose administration until the study end (= last visit) should be reported.

The study monitor will carefully monitor all serious and non-serious adverse events during the entire study. Minimum requirements of data to be recorded are: the nature of the event, duration of AE/SAE (start-end), severity, action taken, outcome and, if appropriate, causality.

For SAEs, the initial report must be followed by full written documentation (narrative summary). The Investigator must document all unexpected SAEs, including the time of onset, complete description of the event, duration, actions taken and outcome.

Adverse events already documented in the CRF at a previous visit and designated as "ongoing" should be reviewed at subsequent visits, as necessary. If these are resolved, the documentation in the CRF should be completed.

Any serious adverse events, whether or not considered to be related to the study vaccine must be reported by the investigator to DICTAGENE by fax within 24 hours (1

calendar day) of becoming aware of the event.

The investigator will document all available information regarding the serious adverse event on the Serious Adverse Event pages contained in the individual Case Report Form and send by fax to Dictagene. The investigator should not wait to receive additional information to fully document the event before notifying the sponsor of a serious adverse event. This initial notification should include, as a minimum, sufficient information to permit identification of:

- the reporter
- the subject
- study vaccine(s)
- adverse event(s)
- date of onset

In the event of a death or a serious adverse event determined by the investigator to be related to vaccination, receipt of the fax must be confirmed by a telephone call.

The initial fax report should be followed by a full summary utilizing the Serious Adverse Event Form, detailing relevant aspects of the adverse events in question. Where applicable, information from relevant hospital case records and autopsy reports should be obtained.

Instances of death, cancer or congenital abnormality in offspring, if brought to the attention of the investigator AT ANY TIME after cessation of the study, should be reported to Dictagene.

Women should be instructed to notify the investigator if it is determined, after completion of the study, that they became pregnant either during the study or within one month (minimum 30 days) after receiving the last vaccine dose. Although not considered an adverse event, pregnancy must be reported on the specific Pregnancy Report Form within 24 hours (1 calendar day) of the investigator's becoming aware of the event. Pregnancy Report Forms are provided in the investigator's study file.

A pregnancy should be followed to term, any premature terminations reported, and the health status of the mother and child, including date of delivery and the child's gender and weight, should be reported to Dictagene after delivery.

The sponsor, DICTAGENE SA, shall inform the Competent Authority of any Serious Adverse Event that have occurred in the study. In addition, any SAE related to the administration of AS02A must be reported by RMF Dictagene to the Head of Clinical Safety of GlaxoSmithKline Biologicals within a maximum of 72 hours after becoming aware of its occurrence.

Study Contact for Reporting Serious Adverse Events	
Name, address:	Dr. Hildur E. BLYTHMAN
Tel:	+ 41 21 785 60 60
	+ 41 21 785 60 20 (direct)
Fax:	+ 41 21 785 60 61

7.0 EVALUATION CRITERIA

7.1 Safety evaluation

Safety of the injected study materials will be determined as the incidence of adverse events during the procedure, and at the intervals indicated in the flowchart above. Volunteers will undergo complete physical examinations, including vital signs (blood pressure, pulse, temperature) and body systems (cardiovascular, gastro-intestinal, CNS, ENT, respiratory, uro-genital, dermatology). Temperature will also be measured daily by the subject and recorded on the diary card provided.

Local solicited adverse events are: redness and/or induration and/or pain and/or reduced mobility of the upper arm(s) up to 72 hours following the injection. They will be measured 1 hour, 24 hours, 48 hours and 72 hours post-injection, and recorded in the Diary Card by the subject and in the appropriate CRF pages by the Investigator.

Systemic solicited adverse events are: erythema, exanthema, urticaria, fatigue, fever, joint pain, myalgia, headache, dyspnea, fainting, dizziness, sweating, nausea, vomiting, diarrhea, palpitations. They will be measured at the same time intervals indicated above, and recorded in the Diary Card and/or CRF pages.

7.2 Immunogenicity evaluation

The criteria for evaluation of immunogenicity of the peptide in combination with the different adjuvants will be the measurement of:

a) the specific anti-P*f* CS 102 antibody response;
b) the recognition of the parasite in a direct immuno-fluorescence test (IFA);
c) the proliferative $CD4^+$ T-cell response to P*f* CS 102 peptides;
d) the production of γ–IFN induced by P*f* CS 102 during the $CD4^+$ proliferation assay.

****

8.0 ANALYSIS OF THE RESULTS

8.1 Data analysis

The data collected with the CRFs will be entered into a database set up by an external Clinical Research Organization, in collaboration with the project statistician.

The data will be analyzed by the project statistician, using SAS statistical software.

The statistical analysis will be performed by the project statistician, in cooperation with an external Clinical Research Organization.

8.2 Criteria for exclusion from analysis

Safety data analyses will be performed on all subjects who received at least one dose of study vaccine: i.e.: *safety population.*

Subjects will be included in the final analysis of *immunogenicity* if they fulfill the following criteria:

1. all inclusion/exclusion criteria were respected
2. they received the vaccine they were randomized to receive
3. they received a second dose of the vaccine
4. allowed intervals between injections were respected
5. allowed intervals between blood sampling schedules were respected
6. no forbidden vaccine or concomitant medication was taken

****

9.0 STATISTICS

9.1 Statistical hypothesis

The P*f* CS 102 synthetic peptide is safe for inoculation into non-immune subjects. No serious adverse events are expected in the sample, other than transient grade 3 (severe) adverse events, similar to those seen previously with experimental vaccines containing either Montanide ISA 720 or AS02A.

The P*f* CS 102 synthetic peptide induces specific immune responses in at least 1/3 of the volunteers.

Assuming neither adjuvant is excluded on safety grounds, the choice of adjuvant for further development will be made by comparing median values and distributions of the measured immunological parameters. The Elispot results for CD8 T-cell levels will be of particular relevance. As is usual for Phase I trials, the study is not powered to ensure that the difference between groups will be statistically significant.

9.2 Sample size calculation

The sample size is determined by the requirement to determine safety of each dosing regimen. The study design cannot ensure that differences in immunogenicity between regimens are statistically significant. Twelve subjects at each vaccine dose constitute a reasonable sample size to estimate the frequency of AEs with an acceptable accuracy, allowing for dropouts.

9.3 Statistical analysis

<u>Safety data</u>: Data will be collected at baseline (V1) and at 4, 10, 28 and 52 weeks will be listed for the study group.

<u>Adverse Events</u>: Descriptive statistics will be used to analyze adverse events (AEs), including intercurrent illnesses, for the study group. The number of AEs and their severity will be assessed. Safety data will be reported using frequency tables of numbers of AEs. With frequently occurring (10 or more) event types, effects of adjuvant and vaccine dose will be tested using logistic regression models, with both the type of adjuvant and vaccine dose as factors in the model. Statistical significance will be assessed by likelihood ratio tests.

<u>Immunogenicity Data</u>: Immunological data for each time point will be analysed separately.

Descriptive statistics of antibody levels determined by the specific anti-P*f* CS 102 antibody titers at baseline and at 4, 8, 10, 26, 28 and 52 weeks.

Descriptive statistics of lymphocyte stimulation indices (SI), as determined by the proliferative $CD4^+$ and $CD8^+$ T-cell response and production of γ-IFN.

Assessment of new responders for humoral and cell-mediated immunity after each immunisation and overall. A "responder" is a subject that has a positive response, in the immunological tests:

a) for <u>antibodies</u>, the *baseline* is the value obtained in naïve subjects (before injections) at a dilution of 1/200. A **positive** antibody response is equal to the baseline + 3 standard deviations of the baseline measurements.

b) for <u>cell-mediated immune responses</u> (CMI) , **positive** responses are as follows:

- $CD4^+$ T-cell proliferation:	S.I. > 3
- $CD8^+$ T cells:	baseline + 3 s.d. for at least one malaria peptide
- γ-IFN production	baseline + 3 s.d.

Comparisons of the response rate for each immune response will be tested separately for each time point using logistic regression models. Both the type of adjuvant and vaccine dose will appear as factors in the model. Statistical significance will be assessed by likelihood ratio tests.

Comparison of logarithmically transformed values of each immune response variable will be carried out using two-way analysis of variance, again, both the type of adjuvant and vaccine dose will appear as factors in the model.

All statistical analyses tests will be performed at an individual significance level of 5% and will be two-sided and will be regarded as exploratory. Therefore no alpha-adjustment for multiplicity is needed.

In addition, all data will be reported in patient listings.

9.4 Interim analysis and final analysis

Data obtained at V7, following the <u>second</u> injection of vaccine, will be collected for an interim analysis. At this time, the performance of the products used for injection will be assessable.

The final analysis will be performed after collection of the 12-months follow-up data for all subjects.

****

10. STUDY MATERIAL

10.1 Packaging, label text and storage

Study material for all treatment groups, will be provided by DICTAGENE SA to the site. The products will be stored in a safe and locked place, with no access to unauthorized personnel, within the Department in which the injections are to be given. Prior to delivery of the study material, the sponsor will inspect the storage area.

One week before each scheduled vaccine injections will take place (day 0, week 8 and week 26) an aliquot of the peptide will be submitted to analytical testing, to verify its stability and chemical properties. In the event of non-conformity, the study will be stopped.

For AS02A, the pre-filled syringes (PFS) must be stored in the refrigerator (+2°C to +8°C) and must not be frozen.

Packages of study materials will be labeled in French.

10.2 Clinical supplies and accountability

DICTAGENE SA will provide the investigator with the clinical trial material; the investigator will follow the instructions as described in Annex C.

On delivery, the investigator will sign receipt of study supplies (injections and documents). A copy of the receipt will be kept in the Investigator's File and the original will be returned to the sponsor. At the end of the study, the sponsor will collect all remaining supplies.

Additional doses of AS02A adjuvant will be provided to replace those that are unusable. In case that an AS02A pre-filled syringe (PFS) is broken or unusable, the vaccinator should use a replacement dose. Although the sponsor need not be notified immediately in these cases, documentation of the use of the replacement dose and reason for using it must be recorded by the investigator on the vaccine accountability form. At the end of the investigation all unused AS02A doses will be returned to GSK within a period of 2 months thereafter.

The sponsor will supply documents that will allow following the dispatch of study material. The investigator will report regularly the actual status to the sponsor.

****

11.0 STUDY AMENDMENT / TERMINATION

11.1 Study amendment

In the event that an **induration** measuring $\geq$ 12 cm in diameter, lasting 48 hours, is observed in **one** subject, lower doses of the vaccine will be used, and the protocol amended accordingly.

11.2 Stopping rule

In the event that any **vaccine-related SAE** is observed, the study may be put on hold pending a full safety review. After discussion with the Investigators, it will be decided whether to proceed with further injections, with equivalent or lower doses, or to terminate the study.

11.3 Go / No-Go criteria for product development

Assuming neither adjuvant is excluded on safety grounds, the choice of adjuvant for further development will be made by comparing median values and distributions of the measured immunological parameters. The Elispot results for CD8 T-cell levels will be of particular relevance. As is usual for Phase I trials, the study is not powered to ensure that the difference between groups will be statistically significant.

Further trials of the product will proceed only if at least 30% of participants have any antibody or cellular responses to the vaccine, with at least one dose- and adjuvant- regimen. The dose and adjuvant of choice for further trials will be selected on the basis of both safety profile and immunogenicity. A regimen which leads to a response in a large proportion of the volunteers will be preferred to one with which the responses are of greater magnitude but present in fewer individuals.

11.4 Study termination

DICTAGENE reserves the right to discontinue the study at any time. The reasons must be discussed with the Principal Investigator; the Ethics Committee and Swissmedic must be informed.

****

12.0 ADMINISTRATIVE ASPECTS

12.1 Financial compensation

All volunteers will receive financial compensation for their participation in the trial, which is intended to cover travel expenses and loss of other financial benefits they may lose by accepting to participate in the trial. Proposed amounts are: CHF 500.00 on week 52 (at visit 11) and CHF 50.00 on week 78 (at visit 12).

12.2 Data collection

The monitoring of this study will be performed by RMF Dictagene.

The study monitor will review the Case Report Forms (CRF), review Serious Adverse Events and/or Unexpected Adverse Events against the source documents.

The data collected in the Case Report Forms (CRF) will include demographic data, results (immunogenicity) and safety data (adverse events). The investigator will sign each page of the CRF.

12.2 Monitoring procedures

The study monitor will ensure the accurate recording of results, the reporting of Adverse Events and Serious Adverse Events. The monitor will also ensure that all aspects of the protocol are followed.

The monitor will also verify that the CRFs are in agreement with the source data. For this purpose, the monitor will be given access to hospital records, original laboratory data, etc., as far as they relate to the study and as agreed with the investigator prior to the study.

The Investigator is to maintain all source documents as required by the protocol, including examination reports, supporting medical records, and Informed Consents. The source documents will be used during the regular monitoring visits to verify information recorded on the Case Report Forms.

12.3 Case Report Forms

Standardized Case Report Forms (CRFs) will be provided for use at the site.

12.4 Quality assurance

Quality assurance procedures are designed to ensure that complete, accurate and timely data are submitted, that protocol requirements are followed, and that complications and adverse events are correctly reported.

Study data will be reviewed by the study monitor to identify inconsistent or missing information and adverse events. Whenever such inconsistent or missing information is found, queries will be sent to the Investigator. All hard copy forms and electronic data files will be secured to ensure confidentiality.

This study will be conducted following GCP guidelines, and may be audited by regulatory authorities, and by the sponsor. The aim is to check compliance with the requirements of GCP guidelines and of this protocol.

****

13.0 REGULATORY AND ETHICAL CONSIDERATIONS

13.1 Declaration of Helsinki, Ethics Committee and Competent Authority

The study will be performed in accordance with the recommendations guiding physicians in biomedical research involving human subjects adopted by the 18th World Association Medical Assembly, Helsinki, Finland, 1964 and later revisions (cf Annex F).

The investigator will obtain approval of the study protocol from the Ethics Committee and keep the EC informed of any Serious Adverse Events and amendments to the protocol. The investigator should file all correspondence with the EC and send the copies to the sponsor.

The sponsor will inform the Competent Authorities of the protocol. The sponsor will assist the investigator to prepare the files which will be submitted to Ethics Committees.

13.2 Subject information and consent

Prior to inclusion in the study, the investigator will give to each subject full and adequate verbal and written information regarding the objectives and the procedures of the study and the possible risks involved. The subjects must be informed about their right to withdraw from the study at any time. Furthermore, it is the responsibility of the investigator to obtain signed informed consent from all subjects prior to inclusion in the study.

All subjects who are entered into the study will have received detailed information regarding the nature of the study materials, the scope of the investigation and the anticipated risks prior to signing the informed consent. A sample patient information letter containing this information can be found in Appendix E.

The information letter and the Informed Consent Form must be approved by the Ethics Committee of the participating site. The original signed consent is retained in the subject's study records, and a copy is provided to the subject.

13.3 Insurance

Any complication related to an expected adverse event, as a consequence of a medical error by the Investigator, will be covered by an insurance taken by the CHUV. Any complication resulting from an unexpected adverse event, related to the antigen provided by the sponsor, will be covered by an insurance taken by RMF Dictagene SA. with Winterthur Assurances. The Certificate N° is 4.526.817/LA.

13.4 Subject data protection

The subjects will be identified in the CRF by a number and initials. The investigator should maintain a subject identification list in the investigator's file, to link medical records and subject numbers used in the CRFs.

The subjects shall be informed that the data will be stored and analyzed by computer, that local regulations for the handling of computerized data will be followed and that identification of individual subject data will only be possible for the investigator and the study monitor. Furthermore, the subjects should be informed about the possibility of inspection of relevant parts of the hospital records by the sponsor and/or Health Authorities.

13.5 Study archives

It is the investigator's responsibility to ensure that the patient identification codes, data source, CRFs and study investigator file are stored securely during the study. Patient files and other source data must be kept for the maximum period of time permitted by the hospital, but not less than 15 (fifteen) years. The sponsor, or subsequent owner, must retain all other documentation pertaining to the trial for the lifetime of the product. Archived data may be stored on microfilm or electronic support, provided that a back-up exists and that a hard copy can be obtained from it, if required.

The protocol, documentation, approvals and all other documents related to the trial, including certificates that satisfactory audit and inspection procedures have been carried out, must be retained by the sponsor in the Trial Master File.

The Final Report must be retained by the sponsor, or subsequent owner, for 5 (five) years beyond the lifetime of the product. Any change of ownership of the data should be documented.

All data and documents will be made available, if requested by relevant authorities.

****

14.0 INVESTIGATORS' RESPONSIBILITIES

14.1 Instruction of study centre staff

It is the investigator's responsibility to ensure that all staff assisting with the study have appropriate qualifications, are fully instructed on the study procedures, and respect the confidentiality statement. The Curriculum Vitae of the Principal Investigator and all co-investigators shall be collected by the study monitor and presented to the Ethics Committees as well as to the Competent Authorities.

14.2 Data recording and adverse event reporting

The CRF should be a complete and accurate record of the subject's data collected during the study, according to GCP recommendations. The investigator is responsible for the quality of the data collected and recorded. He/she will report all adverse events occurring during the study, serious or not, related to the study material or not.

It is the investigator's responsibility to ensure that the data source, CRFs and study investigator file are stored securely during and after the study termination for a period of fifteen years and ensure that such materials are accessible for monitoring, verification and for audits by local officials, DICTAGENE SA and regulatory authorities.

14.3 Protocol deviations

If the medical condition of a subject necessitates a deviation from the protocol, the investigator must contact the sponsor as soon as possible to obtain advice on whether or not the subject is to continue in the study. In the event of an emergency, the Investigator will use his/her medical judgment to resolve the issue. Any protocol deviation must be clearly documented in the CRF, indicating its nature and reasons. Any amendment to the protocol must be discussed and approved by DICTAGENE SA before submission to the Ethics Committee and Regulatory Authorities (Swissmedic).

****

15.0 SIGNATURE SHEET

***PROTOCOL TITLE*:** Phase I safety and immunogenicity trial of a synthetic long peptide (P*f* 282-383 or P*f* CS 102) derived from the circumsporozoite protein of *Plasmodium falciparum,* as a candidate for an anti-malaria vaccine.

***PROTOCOL VERSION*:** Final **DATE: 18/10/2002**

I agree to conduct the study in accordance with the above.

I will ensure that the investigational materials supplied by DICTAGENE will be used as specified in the study protocol.

Principal Investigator ______________________ ______/_____/__________
Dr. François Spertini Date (dd/mm/yy)

Co-Investigator___________________________ ______/_____/__________
Dr. Floriana Lurati Date (dd/mm/yy)

Co-Investigator___________________________ ______/_____/__________
Dr. Cédric Deruaz Date (dd/mm/yy)

Co-Investigator __________________________ _____/_____/___________
Dr. Blaise Genton Date (dd/mm/yy)

Statistician _____________________________ _____/______/__________
Dr. Thomas A. Smith Date (dd/mm/yy)

Annex A

References

1. Clyde, D.F., Most, H., McCarthy, V.C., and Vanderberg, J.P. 1973. Immunization of man against sporozite-induced falciparum malaria. *Am J Med Sci* 266:169-177.
2. Nussenzweig, R.S., Vanderberg, J., Most, H., and Orton, C. 1967. Protective immunity produced by the injection of x-irradiated sporozoites of plasmodium berghei. *Nature* 216:160-162.
3. Rieckmann, K.H., Carson, P.E., Beaudoin, R.L., Cassells, J.S., and Sell, K.W. 1974. Letter: Sporozoite induced immunity in man against an Ethiopian strain of Plasmodium falciparum. *Trans R Soc Trop Med Hyg* 68:258-259.
4. Krzych, U., Lyon, J.A., Jareed, T., Schneider, I., Hollingdale, M.R., Gordon, D.M., and Ballou, W.R. 1995. T lymphocytes from volunteers immunized with irradiated Plasmodium falciparum sporozoites recognize liver and blood stage malaria antigens. *J Immunol* 155:4072-4077.
5. Moreno, A., Clavijo, P., Edelman, R., Davis, J., Sztein, M., Sinigaglia, F., and Nardin, E. 1993. CD4+ T cell clones obtained from Plasmodium falciparum sporozoite-immunized volunteers recognize polymorphic sequences of the circumsporozoite protein. *J Immunol* 151:489-499.
6. Nardin, E.H., and Nussenzweig, R.S. 1993. T cell responses to pre-erythrocytic stages of malaria: role in protection and vaccine development against pre-erythrocytic stages. *Ann. Rev Immunol* 11:687-727.
7. Tsuji, M., Romero, P., Nussenzweig, R.S., and Zavala, F. 1990. CD4+ cytolytic T cell clone confers protection against murine malaria. *J Exp Med* 172:1353-1357.
8. Tsuji, M., Mombaerts, P., Lefrancois, L., Nussenzweig, R.S., Zavala, F., and Tonegawa, S. 1994. Gamma delta T cells contribute to immunity against the liver stages of malaria in alpha beta T-cell-deficient mice. *Proc. Natl. Acad. Sci*. U S A 91:345-349.
9. Potocnjak, P., Yoshida, N., Nussenzweig, R.S., and Nussenzweig, V. 1980. Monovalent fragments (Fab) of monoclonal antibodies to a sporozoite surface antigen (Pb44) protect mice against malarial infection. *J Exp Med* 151:1504-1513.
10. Doolan, D.L., Houghten, R.A., and Good, M.F. 1991. Location of human cytotoxic T cell epitopes within a polymorphic domain of the Plasmodium falciparum circumsporozoite protein. *Int Immunol* 3:511-516.
11. Lalvani, A., Hurt, N., Aidoo, M., Kibatala, P., Tanner, M., and Hill, A.V. 1996. Cytotoxic T lymphocytes to Plasmodium falciparum epitopes in an area of intense and perennial transmission in Tanzania. *Eur J Immunol* 26:773-779.
12. Malik, A., Egan, J.E., Houghten, R.A., Sadoff, J.C., and Hoffman, S.L. 1991. Human cytotoxic T lymphocytes against the Plasmodium falciparum circumsporozoite protein.

Proc. Natl. Acad. Sci. U S A 88:3300-3304.

13. Renggli, J., Valmori, D., Romero, J.F., Eberl, G., Romero, P., Betschart, B., and Corradin, G. 1995. CD8+ T-cell protective immunity induced by immunization with Plasmodium berghei CS protein-derived synthetic peptides: evidence that localization of peptide-specific CTLs is crucial for protection against malaria. *Immunol Lett* 46:199-205.

14. Weiss, W.R., Mellouk, S., Houghten, R.A., Sedegah, M., Kumar, S., Good, M.F., Berzofsky, J.A., Miller, L.H., and Hoffman, S.L. 1990. Cytotoxic T cells recognize a peptide from the circumsporozoite protein on malaria-infected hepatocytes. *J Exp Med* 171:763-773.

15. Ballou, W.R., Hoffman, S.L., Sherwood, J.A., Hollingdale, M.R., Neva, F.A., Hockmeyer, W.T., Gordon, D.M., Schneider, I., Wirtz, R.A., Young, J.F., et al. 1987. Safety and efficacy of a recombinant DNA Plasmodium falciparum sporozoite vaccine. *Lancet* 1:1277-1281.

16. Herrington, D.A., Clyde, D.F., Losonsky, G., Cortesia, M., Murphy, J.R., Davis, J., Baqar, S., Felix, A.M., Heimer, E.P., Gillessen, D., et al. 1987. Safety and immunogenicity in man of a synthetic peptide malaria vaccine against Plasmodium falciparum sporozoites. *Nature* 328:257-259.

17. Brown, A.E., Singharaj, P., Webster, H.K., Pipithkul, J., Gordon, D.M., Boslego, J.W., Krinchai, K., Su-archawaratana, P., Wongsrichanalai, C., Ballou, W.R., et al. 1994. Safety, immunogenicity and limited efficacy study of a recombinant Plasmodium falciparum circumsporozoite vaccine in Thai soldiers. *Vaccine* 12:102-108.

18. Hoffman, S.L., Edelman, R., Bryan, J.P., Schneider, I., Davis, J., Sedegah, M., Gordon, D., Church, P., Gross, M., Silverman, C., et al. 1994. Safety, immunogenicity, and efficacy of a malaria sporozoite vaccine administered with monophosphoryl lipid A, cell wall skeleton of mycobacteria, and squalane as adjuvant. *Am J Trop Med Hyg* 51:603-612.

19. Valmori, D., Pessi, A., Bianchi, E., and Corradin, G. 1992. Use of human universally antigenic tetanus toxin T cell epitopes as carriers for human vaccination. *J Immunol* 149:717-721.

20. Calvo-Calle, J.M., de Oliveira, G.A., Clavijo, P., Maracic, M., Tam, J.P., Lu, Y.A., Nardin, E.H., Nussenzweig, R.S., and Cochrane, A.H. 1993. Immunogenicity of multiple antigen peptides containing B and non-repeat T cell epitopes of the circumsporozoite protein of Plasmodium falciparum. *J Immunol* 150:1403-1412.

21. Migliorini, P., Betschart, B., and Corradin, G. 1993. Malaria vaccine: immunization of mice with a synthetic T cell helper epitope alone leads to protective immunity. *Eur J Immunol* 23:582-585.

22. Romero, P., Maryanski, J.L., Corradin, G., Nussenzweig, R.S., Nussenzweig, V., and Zavala, F. 1989. Cloned cytotoxic T cells recognize an epitope in the circumsporozoite protein and protect against malaria. *Nature* 341:323-326.

23. Hoffman, S.L., Franke, E.D., Hollingdale, M.R., and Druilhe, P. 1996. Attacking the infected hepatocyte, Malaria vaccine development. A multi-immune response approach.:

American Society for Microbiology, Washington D.C. 35-75 pp.

24. Good, M.F., Berzofsky, J.A., Maloy, W.L., Hayashi, Y., Fujii, N., Hockmeyer, W.T., and Miller, L.H. 1986. Genetic control of the immune response in mice to a Plasmodium falciparum sporozoite vaccine. Widespread nonresponsiveness to single malaria T epitope in highly repetitive vaccine. *J Exp Me*d 164:655-660.

25. Good, M.F., Miller, L.H., Kumar, S., Quakyi, I.A., Keister, D., Adams, J.H., Moss, B., Berzofsky, J.A., and Carter, R. 1988. Limited immunological recognition of critical malaria vaccine candidate antigens. *Science* 242:574-577.

26. Riley, E.M., Olerup, O., Bennett, S., Rowe, P., Allen, S.J., Blackman, M.J., Troye-Blomberg, M., Holder, A.A., and Greenwood, B.M. 1992. MHC and malaria: the relationship between HLA class II alleles and immune responses to Plasmodium falciparum. *Int Immunol* 4:1055-1063.

27. Del Giudice, G., Cheng, Q., Mazier, D., Berbiguier, N., Cooper, J.A., Engers, H.D., Chizzolini, C., Verdini, A.S., Bonelli, F., Pessi, A., et al. 1988. Immunogenicity of a non-repetitive sequence of Plasmodium falciparum circumsporozoite protein in man and mice. *Immunology* 63:187-191.

28. Gordon, D.M., McGovern, T.W., Krzych, U., Cohen, J.C., Schneider, I., LaChance, R., Heppner, D.G., Yuan, G., Hollingdale, M., Slaoui, M., et al. 1995. Safety, immunogenicity, and efficacy of a recombinantly produced Plasmodium falciparum circumsporozoite protein-hepatitis B surface antigen subunit vaccine. *J Infect Dis* 171:1576-1585.

29. Sturchler, D., Berger, R., Rudin, C., Just, M., Saul, A., Rzepczyk, C., Brown, G., Anders, R., Coppel, R., Woodrow, G., et al. 1995. Safety, immunogenicity, and pilot efficacy of Plasmodium falciparum sporozoite and asexual blood-stage combination vaccine in Swiss adults. *Am J Trop Med Hyg* 53:423-431.

30. Stoute, J.A., Slaoui, M., Gray-Heppner, D., Momin, P., Kester, K.E., Desmons, P., Wellde, B.T., Garcon, N., Krzych, U., Marchand, M., Ballou, W.R. and Cohen, J.D. 1997. A preliminary evaluation of a recombinant circumsporozoite protein vaccine against Plasmodium falciparum malaria. *N Engl J Med* 336:86-91.

31. Roggero, M.A., Filippi, B., Church, P., Hoffman, S.L., Blum-Tirouvanziam, U., Lopez, J.A., Esposito, F., Matile, H., Reymond, C.D., Fasel, N. and Corradin, G. 1995. Synthesis and immunological characterization of 104-mer and 102-mer peptides corresponding to the N- and C-terminal regions of the Plasmodium falciparum CS protein. *Mol Immunol.* 32:1301-1309.

32. Blum-Tirouvanziam, U., Beghdadi-Rais, C., Roggero, M.A., Valmori, D., Bertholet, S., Bron, C., Fasel, N., and Corradin, G. 1994. Elicitation of specific cytotoxic T cells by immunization with malaria soluble synthetic polypeptides. *J Immunol* 153:4134-4141.

33. Lopez, J.A., Roggero, M.A., Duombo, O., Gonzalez, J.M., Tolle, R., Koita, O., Arevalo-Herrera, M., Herrera, S., and Corradin, G. 1996. Recognition of synthetic 104-mer and 102-mer peptides corresponding to N- and C-terminal nonrepeat regions of the Plasmodium falciparum circumsporozoite protein by sera from human donors. *Am J Trop*

Med Hyg 55:424-429.

34. Lopez, J.A., Gonzalez, J.M., Kettner, A., Arevalo-Herrera, M., Herrera, S., Corradin, G., and Roggero, M.A. 1997. Synthetic polypeptides corresponding to the non-repeat regions from the circumsporozoite protein of Plasmodium falciparum: recognition by human T-cells and immunogenicity in owl monkeys. *Ann Trop Med Parasitol* 91:253-265.

35. Lopez, J.A., Weilenman, C., Audran, R., Roggero, M.A., Bonelo, A., Tiercy, J.M., Spertini, F., and Corradin, G. 2001. A synthetic malaria vaccine elicits a potent CD8(+) and CD4(+) T lymphocyte immune response in humans. Implications for vaccination strategies. *Eur J Immunol* 31:1989-1998.

36. Aucouturier, J., Dupuis, L. and Ganne, V. 2001. Adjuvants designed for veterinary and human vaccines. *Vaccine* 19: 2666-2672.

37. Aucouturier, J., Dupuis, L., Deville, S., Ascarateil, S. and Ganne, V. Montanide ISA 720 and 51: a new generation of water in oil emulsions as adjuvants for human vaccines. *Expert Review of Vaccines* 1 (1), 111 (2002)

38. Saul A, Lawrence G, Smillie A, Rzepczyk CM, Reed C, Taylor D., Anderson, K., Stowers, A., Kemp, R., Allworth, A., Anders, R.F., Brown, G.V., Pye, D., Schoofs, P., Irving, D.O., Dyer, S.L., Woodrow, G.C., Briggs, W.R., Reber, R. and Sturchler, D. 1999. Human phase I vaccine trials of 3 recombinant asexual stage malaria antigens with Montanide ISA720 adjuvant. *Vaccine* 17: 3145-59.

39. Toledo H, Baly A, Castro O, Resik S, Laferte J, Rolo F et al. 2001. A Phase I clinical trial of a multi-epitope polypeptide TAB9 combined with Montanide ISA 720 adjuvant in non-HIV-1 infected human volunteers. *Vaccine* 19, 4328-36.

40. Genton, B., Al-Yaman, F., Anders, R.F., Saul, A., Brown, G., Pye, D., Irving, D.O., Briggs, W.R., Mai, A., Ginny, M., Adiguma, T., Rare, L., Giddy, A., Reber-Liske, R., Stuerchler, D. and Alpers, M.P. 2000. Safety and immunogenicity of a three-component blood-stage malaria vaccine in adults living in an endemic area of Papua New Guinea. *Vaccine*. 18:2504-2511.

41. Genton, B., Betuela, I., Felger, I., Al-Yaman, F., Anders, R.F., Saul, A., Rare, L., Baisor, M., Lorry, K., Brown, G.V., Pye, D., Irving, D.O., Smith, T.A., Beck, H-P. and Alpers, M.P. 2002. A recombinant blood-stage malaria vaccine reduces *Plasmodium falciparum* density and exerts selective pressure on parasite populations in a phase 1-2b trial in Papua New Guinea. *J. Infect. Dis.* 185 : 820-827.

****

Annex B

Abbreviations

ADR:	adverse drug reaction
AE:	adverse event
ALAT :	alanine aminotransferase
ASAT :	aspartate aminotransferase
CHUV:	Centre Hospitalier Universitaire Vaudois
CMI:	cell-mediated immunity
CRF:	Case Report Form
CS:	Circumsporozoite
CTL:	cytotoxic T-lymphocyte
CTX:	clinical trial exemption
ELISA:	enzyme-linked immunosorbent assay
ELISpot:	Enzyme-Linked ImmunoSpot
GCP:	good clinical practice
GLP:	good laboratory practice
GMP:	good manufacturing practice
Hb:	haemoglobin
HBV:	hepatitis B virus
HCV:	hepatitis C virus
HIV:	human immunodeficiency virus
HLA:	human leukocyte antigen
IB:	Investigator's Brochure
ICH:	International Conference on Harmonisation
IFA:	immuno-fluorescence assay
IFN:	interferon
IND:	Investigational New Drug
MCH:	mean corpuscular hemoglobin
MCHC:	mean corpuscular hemoglobin concentration
MCV:	mean corpuscular volume
NaCl:	sodium chloride
NDA:	New Drug Application
PFS:	pre-filled syringe
RBC:	red blood cells
SAE:	serious adverse event
SAS:	Statistical Analysis System
s.d.:	standard deviation
S.I.:	stimulation index
WBC:	white blood cells

Annex C

Methods for the preparation of the vaccines for injection

1.0 Description of material

- P*f* CS 102 samples are available as aliquots of 120 µg lyophilized peptides (DICTAGENE, Lausanne, Switzerland).

- NaCl 0.9% is available in bottles of 10 ml.

- Montanide ISA 720 is an oily adjuvant composition containing a natural metabolizable oil and a highly refined emulsifier based on mannide oleate. It is designed for the production of water-in-oil (W/O) injectable emulsions. It is a ready-to-use product which can be mixed directly with an aqueous medium to provide stable and very fluid formulations. The recommended ratio is 70 % of adjuvant for 30 % of aqueous media (weight/weight). Sterile grade adjuvant is available in 3 ml ampoules (SEPPIC, 75 Quai d'Orsay, 75321 Paris CEDEX 07, France).

- AS02A is an oil-in-water (O/W) formulation, containing the immunostimulants 3D-MPL and QS21. (QS21 is a purified triterpene glycoside from the tree *Quillaja saponaria*). AS02A is available as PFS/aliquots of 600 µl. (GlaxoSmithKline Biologicals SA, Rixensart, Belgium).

2.0 Storage of the peptide and the adjuvants

- P*f* CS 102 is stored in a <u>refrigerator</u>, locked at all times. The refrigerator is equipped with a thermometer and an alarm. The temperature of the refrigerator must be 4°C to 8°C continuously controlled.

  <u>Important notice</u>: One week before each scheduled vaccine preparation, an aliquot of the Pf CS 102 peptide will be submitted to analytical testing, to verify its stability and chemical characteristics.

- Montanide ISA 720 is a clear yellow liquid, <u>stable at room temperature and 4° C</u>. It will be stored in a room which is locked at all times.

- AS02A pre-filled syringes (PFS) must be stored <u>in the refrigerator</u> (+ 2°C to + 8°C) and must <u>not be frozen</u>.

3.0 Preparation of the vaccine with the different adjuvants

The preparation of the P*f* CS 102 vaccine is done under sterile conditions (laminar air flow with sterile gloves).

To inject a volume of 500 µl, it is necessary to prepare a volume with 20% in excess, i.e. a total volume of 600 µl.

3.1 Preparation of the vaccine with Montanide ISA 720:

The peptide vials contain **120 µg** of lyophilized P*f* CS 102. Reconstitute with **120 µl of NaCl 0.9%**. The solution obtained contains **<u>1mg/ml of peptide.</u>** This solution is stable 24 hours after preparation.

For the emulsion, 3 volumes of a peptide solution is mixed with 7 volumes of Montanide ISA 720. Using a pipette, introduce in a sterile recipient, the P*f* CS 102 peptide solution containing 1 mg/ml, plus the NaCl 0.9% and the adjuvant, in the proportions indicated in the table below, to obtain the wanted vaccine dose, then mix using a 10 ml syringe (without rubber) and a 21G needle: push and pull the syringe plunge a minimum of 10 times.

**Vaccine dose (µg of peptides) to be injected**	**10 µg**	**30 µg**	**100 µg**
Volume to be prepared	600 µl	600 µl	600 µl
P*f* CS 102 peptide solution [1 mg/ml]	12 µl	36 µl	120 µl
NaCl 0.9%	168 µl	144 µl	60 µl
Montanide ISA 720	420 µl	420 µl	420 µl
**Final vaccine volume to inject**	**500 µl**	**500 µl**	**500 µl**

Once the vaccine is prepared, it is transported at room temperature in a closed box to the site where the volunteers will be injected.

For the vaccination procedure, inject 0.5 ml, as indicated above, in the intra-muscular tissue in the lateral side of the arm that was not used for blood sampling that day, if possible.

3.2 Preparation of the vaccine with AS02A :

Disinfect the top of a peptide vial (containing **120 µg** of lyophilized P*f* CS 102) with alcohol swabs - let dry. Reconstitute with AS02A, using a pre-filled syringe (PFS) containing 600 µl of adjuvant; inject the AS02A into the vial of lyophilized vaccine. Remove the needle from the vial. The pellet is then dissolved by gently shaking the vial. Wait for 1 minute to ensure complete dissolution of vial contents. The solution obtained contains **200 µg/ml** in AS02A. To withdraw different amounts of the reconstituted vaccine solution, use a fresh needle and syringe each time.

To prepare the different final vaccine concentrations, follow the quantities indicated in the table below:

**Vaccine dose (µg of peptides) to be injected**	**10 µg**	**30 µg**	**100 µg**
Volume to be prepared	600 µl	600 µl	600 µl
P*f* CS 102 peptide solution [200 µg/ml]	60 µl	180 µl	600 µl
AS02A	450 µl	350 µl	0
**Final vaccine volume to inject**	**500 µl**	**500 µl**	**500 µl**

Each 500 µl dose should be administered within 4 hrs of reconstitution by slow intra-muscular injection in the assigned deltoid muscle.

4.0 Vaccination of volunteers :

The vaccinees will be observed closely for at least 30 minutes, with appropriate medical treatment readily available in case of a rare anaphylactic reaction following the administration of vaccines. They will be observed again 48 hours later; they should report any adverse effect during the following 4 days post-injection on a Diary Card provided.

Volunteers will be seen at the Service d'Immunologie & Allergologie, Centre de Vaccinologie of the Hospital Beaumont. For women, a urinary pregnancy test will be performed before each vaccination.

Blood samples will be collected when required by the protocol. All tests will be performed at the CHUV's R&D laboratory of the Immunology & Allergy Service, Hôpital Orthopédique, Av. Pierre Decker 4, Lausanne.

****

Annex D

Definitions Relating To Adverse Events

(Reference: ICH Guideline: Clinical Safety Data Management - Definitions and Standards for Expedited Reporting)

Adverse Event (or Adverse Experience, AE)

Any untoward medical occurrence in a patient or clinical investigation subject administered a pharmaceutical product and which does not necessarily have a causal relationship with the treatment.

Explanation:

An AE can be any unfavourable or unintended sign [including a laboratory finding], symptom or disease temporally associated with the use of a medicinal product, whether or not considered related to the medicinal product.

Adverse Drug Reaction (ADR) [for pre-marketing clinical studies]**:**

All noxious and unintended responses to a medicinal product related to any dose should be considered adverse drug reactions.

Explanation:

The phrase 'responses to a medicinal product' means that a causal relationship between a medicinal product and an AE is at least a reasonable possibility, i.e., the relationship cannot be ruled out.

Adverse Drug Reaction (ADR) [for post-marketing studies]**:**

A response to a drug which is noxious and unintended and which occurs at doses normally used in man for prophylaxis, diagnosis, or therapy of disease or for modification of physical function.

Serious Adverse Event (SAE)

A serious adverse event or reaction is any untoward medical occurrence that at any dose:

- results in death or is life-threatening

- requires in-patient hospitalisation or prolongation of hospitalisation
- results in persistent or significant disability/incapacity
- is a congenital anomaly/birth defect
- other medical event that may jeopardise the patient or may require intervention to prevent one of the other outcomes listed above.

Explanations:

'Life-threatening': refers to an event from which the patient was at risk of death at the time of the event; it does not refer to an event which hypothetically might have caused death if it were more severe.

'Disabling/incapacitating': if the event results in a substantial disruption of the subject's ability to carry out normal life functions. This definition is not intended to include experiences of relatively minor medical significance such as headache, nausea, vomiting, diarrhea, influenza, and accidental trauma (e.g. sprained ankle).

'Hospitalisation': in general, hospitalization signifies that the subject has been detained (usually involving at least an overnight stay) at the hospital or emergency ward for treatment that would not have been appropriate in the physician's office or out-patient setting. Hospitalization for either elective surgery related to a pre-existing condition which did not increase in severity or frequency following initiation of the study or for routine clinical procedures (including hospitalization for "social" reasons) that are not the result of an adverse event, need not be considered as adverse events and are therefore not serious adverse events. When in doubt as to whether 'hospitalization' occurred or was necessary, the adverse event should be considered as serious.

Unexpected Adverse Drug Reaction

An adverse reaction, the nature or severity of which is not consistent with the applicable product information (e.g. the Investigator's Brochure (IB) for an unapproved investigational medicinal product).

Explanation:

Any AE more specific or more severe than that described in the IB should be considered 'unexpected'.

Expedited Reporting

The prompt reporting to regulatory authorities of ADRs that might be significant enough to lead to important changes in the way a medicinal product is developed (e.g. changes in dose, population, monitoring required, or patient information documentation). This is particularly true for ADRs that are life-threatening in their most severe forms.

Explanations:

The usual criterion for expedited reporting is that an ADR should be both serious and unexpected by the definitions above, but other fundamental events should also be the subject of expedited reporting, for example:

- *an increase in the frequency of a serious ADR identified in the protocol as expected.*
- *a significant hazard to the patient population (e.g. lack of efficacy when used in a life-threatening disease)*
- *a major safety finding from a newly completed animal study.*

****

~~Annex~~ E

Sample of the Informed Consent Form

<u>Part 1</u> : Summary of the protocol for the participating volunteers

---

<u>Title of the study</u>: **Phase I safety and immunogenicity trial of a synthetic long peptide (282-383 or P*f* CS 102) derived from the circumsporozoite protein of *Plasmodium falciparum*, as a candidate for an anti-malaria vaccine.**

Dear Madam, dear Sir,

We invite you to participate in a clinical research trial. Before you decide to accept to participate, it is important that you understand why this study is being conducted, what are the implications, and which are the possible benefits, risks or inconveniences related to it. Take your time to read carefully this document and do not hesitate to ask your physician for additional information.

Objectives :

The trial for which we are requesting your participation will test the characteristics of a new vaccine, which is being developed by our company, and which should induce protection against malaria. However, it is still not known if the injection of the vaccine components has any prophylactic effect; therefore, in the event of an exposure to the parasite during a visit to regions endemic for malaria, it is advised to follow the standard prophylactic rules indicated by your physician or your travel agent.

Introduction:

Malaria is the most common of all tropical diseases, and which causes among the highest number of deaths per year, tuberculosis and AIDS being the highest. The estimated number of people exposed to this disease is close to 2,4 billion, and each year, 300.000 – 500.000 individuals become sick. The mortality due to this disease varies between 1.5 – 2.7 million subjects, the vast majority being African children. Other groups with a higher risk of contamination are pregnant women and tourists visiting these regions.

The parasites responsible for this disease belong to the *Plasmodium* species (*P. falciparum, P. vivax, P. malaria, P. ovale*). *P. falciparum* is responsible for the majority of infections and deaths. There is no effective preventive measure which can be implemented in wide geographical areas, therefore, great efforts are being made to develop a vaccine to prevent the disease.

The P*f* CS 102 vaccine:

In a preliminary clinical trial (with human volunteers) it was demonstrated that the P*f* CS 102 protein, derived from *P. falciparum*, could induce an excellent antibody response, as well as an excellent cell-mediated immune response; likewise, it was shown that this vaccine was very well tolerated by the subjects. The aim of this new study is to test a new preparation of the CS 102 protein, associated to “adjuvant” molecules called Montanide and AS02A. If the results of this new trial are positive, the best P*f* CS 102/adjuvant combination will be chosen for another trial, which will have as objective the demonstration of protection after exposure to live parasites.

More than 1000 people have been vaccinated with formulations containing Montanide ISA 51, which is a mineral oil based emulsion, and various publications report the good tolerance of this adjuvant. Montanide ISA 720, based on non-mineral oil, is also in current human clinical trials, and phase I and II demonstrate the good tolerance of the product. The most common effects described were fever, local pain and tenderness; in a few cases, an induration and erythema were also recorded in the placebo (control) group, whereas a number of granulomas and a few sterile abcesses were observed in the vaccine group, and were antigen-dose dependent.

The clinical safety of AS02A-containing vaccines has been assessed in 30 clinical studies comprising prophylactic studies with malaria and HIV-1 vaccines and therapeutic studies with hepatitis B, genital warts, herpes and tumour vaccines. Up to August, 2000, a total of at least 1298 subjects received 3938 doses of AS02A-containing vaccines. The most common local symptom across all studies was injection site pain, sometimes associated with redness, swelling, and/or a transient local muscle soreness and stiffness. None of the reactions required medication, and generally resolved within one week after the injection. The nature and pattern of the symptoms were generally mild to moderate in intensity and resolved without sequelae. Systemic symptoms observed included headache, malaise/fatigue, fever/flu-like symptoms, nausea, vomiting and myalgia. Nearly all grade 3 symptoms probably or suspected to be related to vaccination resolved within 3-7 days.

Study plan:

For this trial we will use a chemically synthesized fragment of the P*f* CS protein. The injection procedure has already been developed by our group. Injections will be done at month 0, 2 and 6. No serious adverse event was observed in prior studies; the only events were mild, and resembled a flu-like state, or local symptoms such as rash, induration, redness, pain at the site of the injection. They were transient, lasting no more than 24 hours.

In this trial we are pursuing two objectives: first, an evaluation of the tolerability to the vaccine. To verify this, a very thorough clinical checkup will be performed, in a hospital environment, following each injection. The second objective is to evaluate the specific immune response to the protein. Different immunological parameters will be measured, in order to evaluate the biological effects of the vaccine, although is will be too early to draw any

conclusions with regard to an eventual protective effect of the vaccine.

The plan is to enroll a total of 36 volunteers, by groups of 6 (each group will receive a different vaccine dose); you will be assigned to one of the 6 groups by an arbitrary number, following a statistical randomisation programme. Your participation in this trial should last 18 months (V12 = week 78).

You cannot have been vaccinated against other diseases during the last 3 months before the first injection, including experimental vaccines against malaria, and you cannot be vaccinated against any other disease until after the 3rd month following the last trial vaccine injection.

In addition, you should not have visited an endemic malaria region during the last 12 months and you will not be allowed to visit, or to stay for any period of time, in a region known to be endemic for malaria. Otherwise you will have to withdraw from the trial.

Please note that you should not perform any physical exercise during the 4 hours preceding and following the vaccine injection and cannot donate your blood during the duration of your participation in this trial.

You will be asked to present yourself for medical checkup and laboratory testing for a total of 12 visits, according to the following timetable:

**Visit Number**	**V1**	**V2**	**V3**	**V4**	**V5**	**V6**	**V7**	**V8**	**V9**	**V10**	**V11**	**V12**
Week	***-1***	***0***	***0***	***4***	***8***	***8***	***10***	***26***	***26***	***28***	***52***	***78***
**Timelines (days)**	- 7	0	2	30	60	62	75	180	182	195	360	540
**Vaccination**		**Inj 1**			**Inj 2**			**Inj 3**				
**Physical examination**	•	•	•		•	•		•	•		•	•
**Blood samples**	•	•		•	•		•	•		•	•	
**Laboratory tests**	•			•			•			•	•	
**Immunology tests**	•	•		•	•		•	•		•	•	

During the first visit a complete physical examination will be performed; blood tests will also be done: a blood sample will be collected (50 ml = a small coffee cup). Similar blood samples will be collected at pre-defined intervals to be able to measure the immune response.

During the first visit, an HIV test will also be done; the results will be given to you, confidentially; a physician will be available for counseling and could follow you therapeutically if you wish. For female volunteers, contraceptive measures will be mandatory

during the first 30 weeks that the study will last, as the eventual effects on the foetus are not yet known. If you are a woman, and you plan to become pregnant (or you become pregnant during the course of the trial) you must inform your physician.

After receiving the injections, you may experience the following 'side effects': pain and/or swelling and/or redness and/or limitation of the movement of the arm where the injection was made; you may also present with fever and/or nausea and/or headache and/or malaise and/or muscle pain and/or fatigue and/or joint pain. Your doctor will examine you and will write down all signs and symptoms observed by him/her and/or reported by you.

As after any vaccination, an acute allergic reaction could be observed: if you feel shortness of breath and/or difficulty to breathe and/or difficulty to swallow and/or dizziness and/or itching, you must inform your physician, who will administer anti-allergic medication, such as anti-histamines or corticosteroids.

Legal considerations

The protocol for this study has been submitted to the Ethics Committee of the CHUV for approval.

The sponsor has subscribed an insurance to cover any unexpected medical eventuality related to the administration of the vaccine studied. Your physician can provide you with information on this matter.

The results obtained during this trial will be kept confidential by anonymizing all files: the sponsor, as well as regulatory authorities and other partner companies can have access to the data collected, but your name will not appear on any document: only your initials and the study code will appear on the documents. In the event that any new information on the study vaccines becomes available, it will be communicated to you.

You are free to interrupt, at any time, your participation in the trial without giving any reason, but you must inform the Investigator of your decision, as a final medical checkup will have to be performed, for your own safety. On the other hand, the sponsor may interrupt the trial at any time; a final medical checkup will be performed.

Your participation in this trial will not cost you anything (all physical and laboratory examinations will not be charged to you); you will receive CHF 550.00 to cover any expenses (travel to the CHUV, eventual loss of revenues). (Proposed amounts are: CHF 500.00 on week 52 and CHF 50.00 on week 78).

For any additional information or any questions you may have, you can contact either Dr. Floriana Lurati (telephone 021.314.08.59) or Dr. François Spertini (telephone 021. 314.07.99) or the Immunology and Allergy Division of the CHUV (switchboard 021.314.11.11, or secretariat 021.314.08.00)

Part 2 ***: Volunteer signature form***

Title of the study: **Phase I safety and immunogenicity trial of a synthetic long peptide (282-383 or P*f* CS 102) derived from the circumsporozoite protein of *Plasmodium falciparum,* as a candidate for an anti-malaria vaccine.**

Before signing this document, please read it carefully.

1. I confirm that I was properly informed of the objectives and the procedures of the above-mentioned trial.

2. I confirm that I received information concerning the details of the study, and had the opportunity to ask all the relevant questions.

3. I confirm that I was given enough time to think about the trial before accepting to participate in it.

4. I confirm that I was informed of the possible risks associated with this trial, as well as the obligations related to the participation in the trial.

5. I agree to provide to the Investigator all information concerning any event, expected or unexpected, taking place during the duration of the trial, and to follow any recommendations given to me by the Investigator.

6. I confirm that I was informed that I can withdraw from the trial at any moment, without any consequences.

7. I confirm that I was informed that the trial may be stopped anytime by the Investigator or by the sponsor, RMF Dictagene, if there are justified reasons for this decision.

8. I agree that all data collected during the course of the study could be communicated to Regulatory Authorities, pharmaceutical companies involved in the development of the compound, but that the confidentiality of the information will be preserved at all times.

9. In addition, I agree that all data collected can be stored by electronic means, in databases for statistical purposes; however, I will have access to these data, by simple request to the Investigator.

10. I confirm that I have indicated to the Investigator that I have not participated in another clinical trial during the month preceding my enrolment into this trial. Likewise, I have been informed that I am not allowed to participate in another clinical trial for the duration of this trial.

11. I confirm that I was informed that the sponsor has subscribed an insurance to cover any event directly related to the vaccine component produced by the sponsor.

12. I confirm that a copy of this informed consent form has been given to me.

**Volunteer's name :**	**Date :**<br>***Signature :***
**Investigator's name :**	**Date :**<br>***Signature :***

Annex F

Declaration of Helsinki

World Medical Association Declaration of Helsinki: Recommendations Guiding Medical Doctors in Biomedical Research Involving Human Subjects.

Adopted by the 18th WMA General Assembly, Helsinki, Finland, 1964
and amended by
the 29th WMA General Assembly in Tokyo, Japan in 1975,
the 35th WMA General Assembly in Venice, Italy in 1983,
the 41st WMA General Assembly in Hong Kong in 1989,
the 48th WMA General Assembly in Somerset West, RSA, October 1996 and
the 52nd WMA General Assembly in Edimburgh, Scotland, October 2000.

A. Introduction

1. The World Medical Association has developed the Declaration of Helsinki as a statement of ethical principles to provide guidance to physicians and other participants in medical research involving human subjects. Medical research involving human subjects includes research on identifiable human material or identifiable data.

2. It is the duty of the physician to promote and safeguard the health of the people. The physician's knowledge and conscience are dedicated to the fulfillment of this duty.

3. The Declaration of Geneva of the World Medical Association binds the physician with the words: "The health of my patient will be my first consideration" and the International Code of Medical Ethics declares that "A physician shall act only in the patient's interest when providing medical care which might have the effect of weakening the physical and mental condition of the patient".

4. Medical progress is based on research which ultimately must rest in part on experimentation involving human subjects.

5. In medical research on human subjects, considerations related to the well-being of the human subject should take precedence over the interests of science and society.

6. The primary purpose of medical research involving human subjects is to improve prophylactic, diagnostic and therapeutic procedures and the understanding of the aetiology and pathogenesis of disease. Even the best proven prophylactic, diagnostic and therapeutic methods must continuously be challenged through research for their effectiveness, efficiency, accessibility and quantity.

7. In current medical practice and in medical research, most prophylactic, diagnostic and therapeutic procedures involve risks and burdens.

8. Medical research is subject to ethical standards that promote respect for all human beings and protect their health and rights. Some research populations are vulnerable and need special protection. The particular needs of the economically and medically disadvantaged must be recognized. Special attention is also required for those who cannot give or refuse consent for themselves, for those who may be subject to giving consent under duress, for those who will not benefit personally from the research and for those for whom the research is combined with care.

9. Research investigators should be aware of the ethical, legal and regulatory requirements for research on human subjects in their own countries as well as applicable international requirements. No national ethical, legal or regulatory requirement should be allowed to reduce or eliminate any of the protections for human subjects set forth in this Declaration.

B. Basic Principles for all Medical Research

10. It is the duty of the physician in medical research to protect the life, health, privacy and dignity of the human subject.

11. Medical research involving human subjects must conform to generally accepted scientific principles, be based on a thorough knowledge of the scientific literature, other relevant sources of information and on adequate laboratory and, where appropriate, animal experimentation.

12. Appropriate caution must be exercised in the conduct of research which may affect the environment, and the welfare of animals used for research must be respected.

13. The design and performance of each experimental procedure involving human subjects should be clearly formulated in an experimental protocol. This protocol should be submitted for consideration, comment, guidance, and where appropriate, approval to a specially appointed ethical review committee, which must be independent of the investigator, the sponsor or any other kind of undue influence. This independent committee should be in conformity with the laws and regulations of the country in which the research experiment is performed. The committee has the right to monitor ongoing trials. The researcher has the obligation to provide monitoring information to the committee, especially any serious adverse events. The researcher should also submit to the committee, for review, information regarding funding, sponsors, institutional affiliations, other potential conflicts of interest and incentives for subjects.

14. The research protocol should always contain a statement of the ethical considerations involved and should indicate that there is compliance with the principles enunciated in this Declaration.

15. Medical research involving human subjects should be conducted only by scientifically qualified persons and under the supervision of a clinically competent medical person. The responsibility for the human subject must always rest with a medically qualified person and never rest on the subject of the research, even though the subject has given consent.

16. Every medical research project involving human subjects should be preceded by careful assessment of predictable risks and burdens in comparison with foreseeable benefits to the subject or to others. This does not preclude the participation of healthy volunteers in medical research. The design of all studies should be publicly available.

17. Physicians should abstain from engaging in research projects involving human subjects unless they are confident that the risks involved have been adequately assessed and can be satisfactorily managed. Physicians should cease any investigation if the risks are found to outweigh the potential benefits or if there is conclusive proof of positive and beneficial results.

18. Medical research involving human subjects should only be conducted if the importance of the objective outweighs the inherent risks and burdens to the subject. This is especially important when the human subjects are healthy volunteers.

19. Medical research is only justified if there is a reasonable likelihood that the populations in which the research is carried out stand to benefit from the results of the results.

20. The subjects must be volunteers and informed participants in the research project.

21. The right of research subjects to safeguard their integrity must always be respected. Every precaution should be taken to respect the privacy of the subject, the confidentiality of the patient’s information and to minimize the impact of the study on the subject's physical and mental integrity and on the personality of the subject.

22. In any research on human beings, each potential subject must be adequately informed of the aims, methods, sources of funding, any possible conflicts of interest, institutional affiliations of the researcher, anticipated benefits and potential risks of the study and the discomfort it may entail. The subject should be informed of the right to abstain from participation in the study or to withdraw consent to participation at any time without reprisal. After ensuring that the subject has understood the information, the physician should then obtain the subject's freely-given informed consent, preferably in writing. If the consent cannot be obtained in writing, the non-written consent must be formally documented and witnessed.

23. When obtaining informed consent for the research project, the physician should be particularly cautious if the subject is in a dependent relationship with the physician or may consent under duress. In that case, the informed consent should be obtained by a well-informed physician who is not engaged in the investigation and who is completely independent of this relationship.

24. For a research subject who is legally incompetent, physically or mentally incapable of giving consent or is a legally incompetent minor, the investigator must obtain informed consent from the legally authorized representative in accordance with applicable law. These groups should not be included in research unless the research is necessary to promote the health of the population represented and this research cannot instead be performed on legally competent persons.

25. When a subject deemed legally incompetent , such as a minor child, is able to give assent to decisions about participation in research, the investigator must obtain that assent in addition to the consent of the legally authorized representative.

26. Research on individuals from whom it is not possible to obtain consent, including proxy or advance consent, should be done only if the physical/mental condition that prevents obtaining informed consent is a necessary characteristic of the research population. The specific reasons for involving research subjects with a condition that renders them unable to give informed consent should be stated in the experimental protocol for consideration and approval of the review committee. The protocol should state that consent to remain in the research should be obtained as soon as possible from the individual or a legally authorized representative.

27. Both authors and publishers have ethical obligations. In publication of the results of research, the investigators are obliged to preserve the accuracy of the results. Negative as well as positive results should be published or otherwise publicly available. Sources of funding, institutional affiliations and any possible conflicts of interest should be declared in the publication. Reports of experimentation not in accordance with the principles laid down in this Declaration should not be accepted for publication.

C. Additional Principles for Medical Research Combined with Medical Care

28. The physician may combine medical research with medical care, only to the extent that the research is justified by its potential prophylactic, diagnostic or therapeutic value. When medical research is combined with medical care, additional standards apply to protect the patients who are research subjects.

29. The benefits, risks, burdens and effectiveness of a new method should be tested against those of the best current prophylactic, diagnostic and therapeutic methods. This does not exclude the use of placebo, or no treatment, in studies where no proven prophylactic, diagnostic or therapeutic method exists.

30. At the conclusion of the study, every patient entered into the study should be assured of access to the best proven prophylactic, diagnostic or therapeutic methods identified by the study.

31. The physician should fully inform the patient which aspects of the care are related to the

research. The refusal of a patient to participate in a study must never interfere with the patient- physician relationship.

32. In the treatment of a patient, where proven prophylactic, diagnostic or therapeutic methods do not exist or have been ineffective, the physician, with informed consent from the patient, must be free to use unproven or new prophylactic, diagnostic or therapeutic measures, if in the physician's judgement it offers hope of saving life, re-establishing health or alleviating suffering. Where possible, these measures should be made the object of research, designed to evaluate their safety and efficacy. In all cases, new information should be recorded and, where appropriate, published. The other relevant guidelines of this Declaration should be followed.

****
