# Supplementary material for: The Synthetic Plasmodium falciparum Circumsporozoite Peptide PfCS102 as a Malaria Vaccine Candidate: A Randomized Controlled Phase I Trial
Source: PLoS One. 2009 Oct 2;4(10):e7304. doi: 10.1371/journal.pone.0007304 (PMC2749339; doi:10.1371/journal.pone.0007304)
Supplement: Approval letter S1 — Ethical Review Board approval letter (0.34 MB PDF) [file pone.0007304.s003.pdf]

UNIVERSITE DE LAUSANNE

**FACULTE DE MEDECINE**
COMMISSION D'ETHIQUE
DE LA RECHERCHE CLINIQUE
RUE DU BUGNON 21-CH-1005 LAUSANNE

PROF. M. BURNIER
PRESIDENT

SECRETARIAT CENTRAL
TEL. +41 21 692 50 08
FAX +41 21 692 50 05

SOUS-COMMISSION I
PRESIDENT PROF. M. BURNIER
TEL : +41 21 345 20 96

SOUS-COMMISSION II
PRESIDENT DR F. GOMEZ, PD, MER
TEL. : +41 21 314 05 96

SOUS-COMMISSION III (PSYCHIATRIE)
PRESIDENT DR J. GASSER, PD, MER
TEL. : +41 21 643 64 65

Prof. F. Spertini
Professeur associé
Service d'immunologie et
d'allergie
CHUV
1011 Lausanne

Lausanne, le 24 octobre 2002

## Avis de la Commission d'Ethique de la recherche clinique

Monsieur et cher Collègue,

Lors de sa séance du **9 septembre 2002**, la Commission d'Ethique de la recherche clinique, Sous-Commission I (dans la composition détaillée en page 4) a procédé à une évaluation approfondie du projet de recherche désigné ci-après :

**Protocole 162/02 :** Etude clinique de phase I, sur la tolérance et l'immunogénicité d'un long peptide synthétique (282-383 ou *Pf*CS 102) dérivé de la protéine circumsporozoaire du *Plasmodium falciparum* comme candidat en tant que vaccin antipaludique

**Investigateur :**

Prof. F. Spertini
Professeur associé
Service d'immunologie et
d'allergie
CHUV
1011 Lausanne

La Commission d'Ethique de la recherche clinique, Sous-Commission I, base son appréciation sur les documents soumis les 26.08.2002 et 16.10.2002, soit :

- Brochure de l'investigateur, version en anglais No 1 du 27.08.2002
- Protocole en français, version finale du 26.08.2002 (corrigée le 18 octobre 2002)
- Formulaire d'information et de consentement éclairé et écrit, version finale du 28.08.02
- Formulaire de recueil de Consentement éclairé, à signer par le sujet volontaire du 28.08.02, corrigé le 18.10.2002
- Attestation d'assurance du 15.08.2002
- Curriculum vitae
- Mode de rémunération du 27.08.2002
- Affichette
- Cahier d'observations (recueil des données) des sujets de recherche
- Journal (carnet de Bord) des sujets de recherche modifié
- Votre lettre du 16.10.2002

☒ procédure ordinaire ☐ procédure simplifiée ☐ évaluation ultérieure

La Commission d'Ethique arrête l'**avis** suivant :

☒ A **Avis positif**
☐ B **Avis positif assorti de recommandations** (v. page 3 et suiv.)
Information écrite à la Commission d'éthique suffisante ☐
☐ C **Avis conditionnel** (v. page 3 et suiv.)
Evaluation ultérieure par la Commission d'éthique nécessaire ☐
Information écrite à la Commission d'éthique suffisante ☐
☐ D **Avis négatif motivé (et explication pour réexamen)** (v. page 3 et suiv.)
☐ E **Avis justifié de ne pas entrer en matière** (v. page 3 et suiv.)

L'avis s'applique également aux autres investigateurs mentionnés dans la demande d'évaluation, travaillant dans des sites de recherche relevant du champ de compétence de la Commission d'Ethique de la recherche clinique.

Pour mémoire : **Obligations de l'investigateur**

- Les produits testés et de comparaison (médicaments et dispositifs médicaux) doivent être fabriqués, évalués et utilisés conformément aux règles de l'art visant à en garantir la qualité et la sécurité.
- Devoir de signaler: a) immédiatement tout événement indésirable grave (serious adverse events)
  b) toute information devenant disponible au cours de l'essai et ayant des conséquences directes pour la sécurité des sujets et la poursuite de l'essai
  c) Modification du protocole
  d) Fin ou arrêt prématuré de l'essai
- Rapport intermédiaire: une fois par année
- Obligation de notifier les essais ou de demander l'autorisation correspondante auprès de Swissmedic ou d'autres autorités fédérales ou cantonales (en cas d'étude sponsorisée, cette tâche incombe au promoteur)
- Rapport final

**Emolument :** Fr. 800.--

**La Commission d'éthique, Sous-Commission I :**

Prof. Michel Burnier
Président

**FORMULAIRE DE RAPPORT INTERMEDIAIRE/FINAL**
**pour la Commission d'éthique de la recherche clinique**
**Sous-Commission I**

**Investigateur :**

Prof. F. Spertini
Professeur associé
Service d'immunologie et
d'allergie
CHUV

**Protocole 162/02 :** Etude clinique de phase I, sur la tolérance et l'immunogénicité d'un long peptide synthétique (282-383 ou PfCS 102) dérivé de la protéine circumsporozoaire du *Plasmodium falciparum* comme candidat en tant que vaccin antipaludique

**Etude terminée:** OUI ❑ NON ❑ **Etude en cours:** OUI ❑ NON ❑

**Etude arrêtée:** OUI ❑ NON ❑

**Si OUI, pourquoi?**

..............................................................................................................................
..............................................................................................................................
..............................................................................................................................

**Conclusions qui peuvent être tirées de l'étude à ce stade:**

..............................................................................................................................
..............................................................................................................................
..............................................................................................................................
..............................................................................................................................
..............................................................................................................................

**Y a-t-il eu des incidents pendant l'étude?** OUI ❑ NON ❑

**Si OUI, lesquels?**

..............................................................................................................................
..............................................................................................................................
..............................................................................................................................

**Fin de l'étude prévue pour:** ..............................................................................................

---

**A retourner au:** Prof. M. Burnier
Président de la Commission d'Ethique de la Recherche clinique de la Faculté de Médecine
Rue du Bugnon 21, 1005 Lausanne

En cas de publication, prière d'adresser un tiré-à-part.

---

**Date de l'acceptation de l'étude par la Commission d'Ethique:** 24.10.2002
**Date limite pour le renvoi de ce formulaire:** 24.10.2003

# Séance du 9 septembre 2002

## Composition de la Commission d'éthique de la recherche clinique, Sous-Commission I

L'avis de la Commission d'éthique ayant siégé dans sa composition détaillée ci-après est valable, le quorum étant atteint (art. 32 de l'Ordonnance sur les essais cliniques de produits thérapeutiques du 17 octobre 2001).

| | | | | | participe à l'avis | |
|---|---|---|---|---|---|---|
| | **Nom, prénom** | **Profession, titre** | H | F | **oui** | **non** |
| **Présidence** | Burnier Michel | Professeur associé | ☒ | ☐ | ☒ | ☐ |
| **Membres** | Baudraz Marie-Claire | Médecin installé, spéc. FMH | ☐ | ☒ | ☒ | ☐ |
| | Boillat Marcel-André | Professeur ordinaire | ☒ | ☐ | ☒ | ☐ |
| | Foppa Carlo | Docteur, PhD, Ethicien | ☒ | ☐ | ☒ | ☐ |
| | Gailloud Claude | Professeur honoraire | ☒ | ☐ | ☒ | ☐ |
| | Gardaz Jean-Patrice | Docteur, PD, MER | ☒ | ☐ | ☒ | ☐ |
| | Lejeune Ferdy | Professeur ordinaire | ☒ | ☐ | ☒ | ☐ |
| | Joye Charles | Avocat | ☒ | ☐ | ☒ | ☐ |
| | Pannatier André | Professeur en pharmacie | ☒ | ☐ | ☐ | ☒ |
| | Reymond Catherine | Infirmière HMP | ☐ | ☒ | ☒ | ☐ |
| | Rossel Martine | Infirmière enseignante | ☐ | ☒ | ☒ | ☐ |
| Membre compétent en biométrie | Burnand Bernard | Docteur, PD, MER | ☒ | ☐ | ☐ | ☒ |
