# Supplementary material for: The Synthetic Plasmodium falciparum Circumsporozoite Peptide PfCS102 as a Malaria Vaccine Candidate: A Randomized Controlled Phase I Trial
Source: PLoS One. 2009 Oct 2;4(10):e7304. doi: 10.1371/journal.pone.0007304 (PMC2749339; doi:10.1371/journal.pone.0007304)
Supplement: Approval letter S2 — Swissmedic approval letter (0.91 MB PDF) [file pone.0007304.s004.pdf]

Confirmation de notification
pour les essais cliniques de médicaments

(à remplir par le promoteur)

1.	Promoteur / adresse	RMF Dictagene SA - Chemin de la Vulliette 4 - 100 Lausanne 25
1a.	Personne en Suisse désignée au sens de l'art. 7 de l'Ordonnance (Oclin) / adresse	Winterthur Assurances<br>Ru Benjamin-Constant 1 - 1200 Lausanne
1b.	Organisme de recherche (CRO)	MedPass International
2.	Investigateur responsable / adresse	Dr. F. Spertini - Serv. d'Immunologie et d'Allergie - CHUV -<br>Rue du Bugnon - 1011 Lausanne
2a.	Autres investigateurs responsables, liste mise à jour (datée)	Voir Liste annexe.
3.	Essai clinique / titre / N° du protocole	Etude clinique de phase I, sur la tolérance et l'immunogenicité d'un long peptide synthétique (282-383 ou PfCS102) dérivé de la protéine circumsporozoaire du Plasmodium falciparum, comme candidat en tant que vaccin anti-paludique. [DG001]
4.	Médicament d'essai	PfCS102

A laisser en blanc!

Numéro de référence de l'essai clinique: 2002 DR 1 2 2 7

Il est confirmé que l'essai clinique décrit a été enregistré à l'Institut suisse des produits thérapeutiques. L'essai clinique peut commencer dès réception de cette confirmation de notification (art. 15, al. 2 de l'Ordonnance sur les essais cliniques de produits thérapeutiques, OClin du 17.10.2001).

swissmedic
Schweizerisches Heilmittelinstitut
Institut suisse des produits thérapeutiques
Istituto svizzero per gli agenti terapeutici
Swiss Agency for Therapeutic Products

swissmedic

Formulaire de notification
pour les essais cliniques de médicaments

Berne, 24 OCT. 2002

Dr. med. Y. Chautems
