# Supplementary material for: The Synthetic Plasmodium falciparum Circumsporozoite Peptide PfCS102 as a Malaria Vaccine Candidate: A Randomized Controlled Phase I Trial
Source: PLoS One. 2009 Oct 2;4(10):e7304. doi: 10.1371/journal.pone.0007304 (PMC2749339; doi:10.1371/journal.pone.0007304)
Supplement: Consent Form S1 — Consent form (0.06 MB PDF) [file pone.0007304.s005.pdf]

Sample of the Informed Consent Form

<u>Part 1</u> : Protocol Summary for the participating volunteers

<u>Title of the study</u>: **Phase I safety and immunogenicity trial of a synthetic long peptide (282-383 or P*f* CS 102) derived from the circumsporozoite protein of *Plasmodium falciparum,* as a candidate for an anti-malaria vaccine.**

Objectives :

The trial for which we are requesting your participation will test the characteristics of a new vaccine, which is being developed by our company, and which should induce protection against malaria. However, it is still not known if the injection of the vaccine components has any prophylactic effect; therefore, in the event of an exposure to the parasite during a visit to regions endemic for malaria, it is advised to follow the standard prophylactic rules indicated by your physician or your travel agent.

Introduction:

Malaria is the most common of all tropical diseases, and which causes the second highest number of deaths per year, tuberculosis being the highest. The estimated number of people exposed to this disease is close to 2,4 billion, and each year, 300.000 – 500.000 individuals become sick. The mortality due to this disease varies between 1.5 – 2.7 million subjects, the vast majority being African children. Other groups with a higher risk of contamination are pregnant women and tourists visiting these regions.

The parasites responsible for this disease belong to the *Plasmodium* species (*P. falciparum, P. vivax, P. malaria, P. ovale*). P. falciparum is responsible for the majority of infections and deaths. There is no effective preventive measure that can

be implemented in wide geographical areas, therefore, great efforts are being made to develop a vaccine to prevent the disease.

The PfCS102 vaccine:

In a preliminary clinical trial (with human volunteers) it was demonstrated that the P*f* CS 102 protein, derived from *P. falciparum*, could induce an excellent antibody response, as well as an excellent cell-mediated immune response; likewise, it was shown that this vaccine was very well tolerated by the subjects. The aim of this new study is to test a new preparation of the CS 102 protein, associated to “adjuvant” molecules called Montanide and AS02. (Adjuvants are substances capable of amplifying the immune response). If the results of this new trial are positive, the best P*f* CS 102/adjuvant combination will be chosen for another trial, which will have as objective the demonstration of protection after exposure to live parasites.

Study plan:

For this trial we will use a chemically synthesized fragment of the P*f* CS protein. The injection procedure has already been developed by our group. Injections will be done at month 0, 2 and 6. No serious adverse event was observed in prior studies; the only events were mild, and resembled a flu state, or local symptoms such as rash, induration, redness, pain at the site of the injection. They were transient, lasting no more than 24 hours.

In this trial we are pursuing two objectives: first, an evaluation of the <u>tolerability</u> to the vaccine. To verify this, a very thorough clinical checkup will be performed, in a hospital environment, following each injection. The second objective is to evaluate the <u>specific immune response</u> to the protein. Different immunological parameters will be measured, in order to evaluate the biological effects of the vaccine, although is will be too early to draw any conclusions with regard to an eventual protective effect of the vaccine.

The plan is to enroll a total of 36 volunteers, by groups of 6 (each group will receive a different vaccine dose); you will be assigned to one of the 6 groups by an arbitrary number, following a statistical randomization programme. The total duration of the trial should be of 18 months.

During the first visit a complete physical examination will be performed; blood tests will also be done: a blood sample will be collected (50 ml = a small coffee cup). Similar blood samples will be collected at pre-defined intervals (at weeks 4, 10 , 26, 28 and 52) to be able to measure the immune response.

Please note that you should not perform any physical exercise during the 4 hours preceding blood testing and cannot donate your blood during the duration of your participation in this trial.

During the first visit, an HIV test will also be done; the results will be given to you, confidentially; a physician will be available for counseling and could follow you therapeutically if you wish. For women of childbearing potential, contraceptive measures will be mandatory during the first 30 weeks that the study will last, as the eventual effects on the foetus are not yet known. If you are a woman, and you plan to become pregnant (or you become pregnant during the course of the trial) you must inform your physician.

You cannot have been vaccinated against other diseases during the last 3 months before the first injection, and you cannot be vaccinated against any other disease until after the 3rd month following the last trial vaccine injection.

In addition, you should not have visited an endemic malaria region during the last 6 months and you will not be allowed to visit, or to stay for any period of time, in a region known to be endemic for malaria. Otherwise you will have to withdraw from

the trial.

You will be asked to present yourself for medical checkup and laboratory testing for a total of 12 visits, according to the following timetable:

**Visit Number**<br><br>***Week***	**V1**<br><br>***-1***	**V2 + 3**<br><br>***0***	**V4 + 5**<br><br>***4***	**V6**<br><br>***8***	**V7**<br><br>***10***	**V8 + 9**<br><br>***26***	**V10**<br><br>***28***	**V11**<br><br>***52***	**V12**<br><br>***78***
**Timelines (days)**	- 7	0 + 2	30 + 3	60	75	180 + 182	195	360	540
**Vaccination**		Inj 1		Inj 2		Inj 3			

After receiving the injections, you may experience the following ‘side effects’: pain and/or swelling and/or redness at the injection site; you may also present with fever and/or nausea and/or headache and/or malaise and/or muscle pain and/or fatigue and/or joint pain and/or limitation of the movement of the arm where the injection was made. Your doctor will examine you and will write down all signs and symptoms observed by him/her and/or reported by you.

In some extremely rare cases, an acute allergic reaction has been observed in previous studies: if you feel shortness of breath and/or difficulty to breathe and/or difficulty to swallow and/or dizziness and/or itching, you must informed your physician, who will administer anti-allergic medication, such as anti-histamines or corticosteroids.

The sponsor has subscribed an insurance to cover any unexpected medical eventuality related to the administration of the vaccine studied. Your physician can provide you with information on this matter.

The results obtained during this trial will be kept confidential by anonymizing all files: the sponsor, as well as regulatory authorities can have access to the data collected, but your name will not appear on any document: only your initials and the study code will appear on the documents.

You are free to interrupt, at any time, your participation in the trial without giving any reason, but you must inform the Investigator of your decision, as a final medical checkup will have to be performed, for your own safety.

Your participation in this trial will not cost you anything (all physical and laboratory examinations will not be charged to you); you will receive CHF 550.00 to cover any expenses (travel to the CHUV, eventual loss of revenues). (Proposed amounts are: CHF 500.00 on week 52 and CHF 50.00 on week 78).

****

For any additional information or any questions you may have, you can contact either Dr. Floriana Lurati (telephone 021.314.39.79) or Dr. François Spertini (telephone 021. 314.07.90) or the Immunology and Allergy Division of the CHUV (switchboard 021.314.11.11, or secretariat 021.314.08.00)

Sample of the Informed Consent Form

Part 2 : Volunteer signature form

Title of the study: **Phase I safety and immunogenicity trial of a synthetic long peptide (282-383 or P*f* CS 102) derived from the circumsporozoite protein of *Plasmodium falciparum,* as a candidate for an anti-malaria vaccine.**

The undersigned :……………………………………………………………………………..

Confirms that he/she was properly informed of the objectives and the procedures of the above-mentioned trial

Confirms that he/she received information concerning the details of the study, and had the opportunity to ask all the relevant questions

Confirms that he/she was informed of the possible risks and eventual advantages associated with this trial, as well as the obligations related to the participation in the trial

Confirms that he/she was given enough time to think about the trial before accepting to participate in it.

Confirms that he/she was informed that he/she could withdraw from the trial at any moment, without any consequences.

Agrees that all data collected during the course of the study could be communicated to Regulatory Authorities, pharmaceutical companies involved in the development of the compound) but that the confidentiality of the information will be preserved at all times.

Agrees to provide to the Investigator all information concerning any event, expected or unexpected, taking place during the duration of the trial, and to follow
